# Identification of Human *N*-Myristoylated Proteins from Human Complementary DNA Resources by Cell-Free and Cellular Metabolic Labeling Analyses

**DOI:** 10.1371/journal.pone.0136360

**Published:** 2015-08-26

**Authors:** Emi Takamitsu, Motoaki Otsuka, Tatsuki Haebara, Manami Yano, Kanako Matsuzaki, Hirotsugu Kobuchi, Koko Moriya, Toshihiko Utsumi

**Affiliations:** 1 Applied Molecular Bioscience, Graduate School of Medicine, Yamaguchi University, Yamaguchi, 753–8515, Japan; 2 Department of Biological Chemistry, Faculty of Agriculture, Yamaguchi University, Yamaguchi, 753–8515, Japan; 3 Department of Cell Chemistry, Okayama University Graduate School of Medicine, Dentistry and Pharmaceutical Sciences, Okayama, 700–8558, Japan; University of Alabama at Birmingham, UNITED STATES

## Abstract

To identify physiologically important human *N*-myristoylated proteins, 90 cDNA clones predicted to encode human *N*-myristoylated proteins were selected from a human cDNA resource (4,369 Kazusa ORFeome project human cDNA clones) by two bioinformatic *N*-myristoylation prediction systems, NMT-The MYR Predictor and Myristoylator. After database searches to exclude known human *N*-myristoylated proteins, 37 cDNA clones were selected as potential human *N*-myristoylated proteins. The susceptibility of these cDNA clones to protein *N*-myristoylation was first evaluated using fusion proteins in which the *N*-terminal ten amino acid residues were fused to an epitope-tagged model protein. Then, protein *N*-myristoylation of the gene products of full-length cDNAs was evaluated by metabolic labeling experiments both in an insect cell-free protein synthesis system and in transfected human cells. As a result, the products of 13 cDNA clones (FBXL7, PPM1B, SAMM50, PLEKHN, AIFM3, C22orf42, STK32A, FAM131C, DRICH1, MCC1, HID1, P2RX5, STK32B) were found to be human *N*-myristoylated proteins. Analysis of the role of protein *N*-myristoylation on the intracellular localization of SAMM50, a mitochondrial outer membrane protein, revealed that protein *N*-myristoylation was required for proper targeting of SAMM50 to mitochondria. Thus, the strategy used in this study is useful for the identification of physiologically important human *N*-myristoylated proteins from human cDNA resources.

## Introduction

Protein *N*-myristoylation is the attachment of myristic acid, a 14-carbon saturated fatty acid, to the *N*-terminal Gly of proteins [1–5]. This modification is one of the major forms of lipid modification that occurs on eukaryotic and viral proteins. It is estimated that approximately 0.5–1.5% of eukaryotic proteins are *N*-myristoylated. In general, myristic acid is cotranslationally attached to the *N*-terminal Gly residue after removal of the initiating Met. In addition to cotranslational protein *N*-myristoylation, it has been established that posttranslational *N*-myristoylation can also occur on many caspase-cleavage products in apoptotic cells [6–8]. Both cotranslational and posttranslational *N*-myristoylation are catalyzed by *N*-myristoyltransferase (NMT), a member of the GCN5-related *N*-acetyltransferase superfamily of proteins [9]. The precise substrate specificity of this enzyme has been characterized using purified enzyme and synthetic peptide substrates [10, 11]. The requirement for Gly at the *N*-terminus is absolute and no other amino acid can take its place.

Many *N*-myristoylated proteins play key roles in regulating cellular structure and function. They include proteins involved in a wide variety of cellular signal transduction pathways, such as protein kinases, phosphatases, guanine nucleotide binding proteins, Ca^2+^ binding proteins, and cytoskeletal regulatory proteins [1–5]. In addition to proteins involved in cellular signal transduction pathways, recent studies have revealed that protein *N*-myristoylation occurs on many disease-related proteins [12–16]. However, comprehensive identification of human *N*-myristoylated proteins has not been achieved because of the lack of a simple and easy strategy to detect protein *N-*myristoylation.

Recent progress in chemical biology has made novel approaches available for the study of protein *N*-myristoylation by taking advantage of bioorthogonal reactions [17, 18]. The advantage of these approaches is that they are non-radioactive, have short detection times and a high degree of sensitivity in comparison with traditional radiolabeling. In fact, metabolic labeling of cellular proteins with bioorthogonal myristic acid analogues and subsequent ligation with secondary reporters enables visualization, enrichment, and MS-based identification of *N*-myristoylated proteins [19, 20]. In addition to these protein-based strategies, human *N*-myristoylated proteins could be identified by cell-free and cellular metabolic labeling using human cDNA clones. In our previous study, to identify novel human *N-*myristoylated proteins, the susceptibility of human cDNA clones from human cDNA resources to protein *N-*myristoylation was evaluated by metabolic labeling and mass spectrometric analyses of proteins expressed using an insect cell-free protein synthesis system [21]. As a result, the products of 18 out of ~2,000 cDNA clones (Kazusa ORFeome project human cDNA clones) were found to be novel *N-*myristoylated proteins that had not been reported previously. These results indicated that this strategy is useful for the identification of human *N-*myristoylated proteins from human cDNA resources.

In this study, to identify physiologically important human *N*-myristoylated proteins, cell-free- and cellular metabolic labeling experiments coupled with bioinformatic prediction systems for protein *N*-myristoylation were performed using 4,369 Kazusa ORFeome project human cDNA clones.

## Materials and Methods

### Materials

Transdirect insect cell, an insect cell-free protein synthesis system, was obtained from Shimadzu (Kyoto, Japan). Human cDNAs (Flexi ORF clones) were purchased from Promega (Madison, WI, USA). [^3^H]leucine, [^3^H]myristic acid, and ECL prime western blotting detection reagent were from GE Healthcare (Buckinghamshire, UK). ENLIGHTNING was from PerkinElmer (Waltham, MA, USA). T7-Scribe standard RNA IVT kit was from CELLSCRIPT (Madison, WI, USA). The dye terminator cycle sequencing kit, Lipofectamine LTX and Plus reagent, MitoTracker Red CMXRos and Hoechst 33342 were from Life Technologies Corporation (Carlsbad, CA, USA). Anti-FLAG monoclonal antibody, anti-SAMM50 monoclonal antibody (WH0025813), anti-SAMM50 polyclonal antibody (HPA034537) and anti-Rabbit IgG-FITC antibody were from Sigma (St. Louis, MO, USA). Protein G-HRP conjugate was from Bio-Rad (Hercules, CA, USA). ImmunoStar LD was from Wako Pure Chemical (Osaka, Japan). X-ray film was from Eastman Kodak (Rochester, NY, USA). The other reagents used were from Wako Pure Chemical (Osaka, Japan), Daiichi Pure Chemicals (Tokyo, Japan) or Seikagaku Kogyo (Tokyo, Japan) and were of analytical or DNA grade.

### Prediction of protein *N*-myristoylation using prediction programs

Two public WWW-server-based prediction programs for protein *N*-myristoylation, MYR Predictor (http://mendel.imp.ac.at/myristate/SUPLpredictor.htm) [22] and Myristoylator (http://www.expasy.org/tools/myristoylator/) [23], were used for the prediction of protein *N*-myristoylation. The entire amino acid sequences deduced from the nucleotide sequences of the ORFs were used as the query.

### Plasmid construction

Nucleotide sequences of oligonucleotide primers used for plasmid construction are summarized in S1 Table. Plasmid pTD1 (Shimadzu) was used for the insect cell-free protein synthesis system [24]. Plasmids pcDNA3-tActin-FLAG, pcDNA3-tActinG2A-FLAG, pTD1-tActin-FLAG, pTD1-tActinG2A-FLAG and pcDNA3-FLAG were constructed as described previously [7, 25]. Construction of the expression vector pTD1-Δ10aa-tActin-Flag for screening *N*-myristoylated proteins is summarized in S2 Table. pTD1 plasmids containing the cDNAs coding for the tActin fusion proteins with *N*-terminal ten amino acid sequences of the ORF of the KOP cDNA clones at the *N*-terminus were constructed as follows. The two oligonucleotides, 5’-ATCNNNNNNNNNNNNNNNNNNNNNNNNNG-3’ (sense strand) and 5’-AATTCNNNNNNNNNNNNNNNNNNNNNNNNNGAT-3’ (antisense strand), coding for the N-terminal 10 amino acid sequence of the ORF of the KOP cDNA clones were annealed. The annealed dsDNAs were individually ligated into EcoRV-EcoRI sites of the pTD1-Δ10aa-tActin-FLAG vector. The resulting plasmids were designated as pTD1-NNNNN-tActin-FLAG, where NNNNN indicates the number of the product ID of the KOP cDNA clones. The construction of the pTD1 or pcDNA3 plasmids including full-length KOP cDNA clones are summarized in S2 Table.

### *In vitro* transcription and translation reaction

mRNAs encoding the cDNAs were prepared using a T7-scribe standard RNA IVT kit (CELLSCRIPT) in accordance with the manufacturer’s instructions. The synthesized mRNAs were purified by phenol-chloroform extraction and ethanol precipitation before use in the translation reaction.

### Cell-free protein synthesis

The translation reaction was performed using an insect cell-free protein synthesis system (Shimadzu) in the presence of [^3^H]leucine or [^3^H]myristic acid as described previously [25]. The mixture (composed of 6.2 μL insect cell lysate, 3.7 μL reaction buffer, 0.5 μL 4 mM methionine, 1.0 μL [^3^H]leucine [1 μCi] or 3.0 μL [^3^H]myristic acid [20 μCi], and 2 μL mRNA [8 μg]) was incubated at 26°C for 6 h. The translation products were then analyzed by SDS-PAGE and fluorography.

### Transfection of cells

HEK293T (a human embryonic kidney cell line) cells or COS-1 (simian virus 40-transformed African green monkey kidney cell line, American Type Culture Collection) cells were maintained in Dulbecco’s modified Eagle’s medium (DMEM; Gibco BRL [Palo Alto, CA, USA]) supplemented with 10% fetal calf serum (FCS; Gibco BRL). Cells (2 × 10^5^) were plated onto 35-mm diameter dishes 1 day before transfection. pcDNA3 constructs (2 μg) containing cDNAs encoding FLAG-tagged proteins were used to transfect the cells in each plate along with 2.5 μL Lipofectamine LTX and 2 μL Plus reagent in 1 mL serum-free medium. After incubation for 5 h at 37°C, the cells were re-fed with serum-containing medium and incubated again at 37°C for appropriate periods.

### Metabolic labeling of cells

The metabolic labeling of cells with [^3^H]myristic acid was performed as described previously [26]. HEK293T cells (2 × 10^5^) were transfected with pcDNA3 constructs (2 μg) containing cDNAs, as described above, and incubated at 37°C for 24 h. Then, they were washed once with 1 mL serum-free DMEM and incubated for 6 h at 37°C in 1 mL DMEM (+2% FCS) containing [^3^H]myristic acid (100 μCi/mL). Subsequently, the cells were washed three times with Dulbecco’s phosphate-buffered saline (DPBS), harvested and lysed with 200 μL of RIPA buffer (50 mM Tris-HCl (pH 7.5), 150 mM NaCl, 1% Nonidet P-40, 0.5% sodium deoxycholate, 0.1% SDS, protease inhibitors) on ice for 20 min. Subsequently, the samples were analyzed by SDS-PAGE and fluorography.

### SDS-PAGE and fluorography

The radiolabeled proteins were resolved by 12.5% SDS-PAGE, then the gel was fixed and soaked in ENLIGHTNING (PerkinElmer) for 20 min. Thereafter, the gel was dried under vacuum and exposed to X-ray film for an appropriate period.

### Western blotting

Proteins were resolved by 12.5% SDS-PAGE and then transferred to an Immobilon-P transfer membrane. After blocking with non-fat milk, the membrane was probed with a primary antibody, as described previously [27]. Immunoreactive proteins were detected specifically by incubation with protein G-HRP conjugate. The membrane was developed using ECL Prime western blotting detection reagent or ImmunoStar LD and detected using a MicroChemi Chemiluminescence Imaging System. The blots were quantified by densitometry using the software TotalLab Quant.

### Immunofluorescence analysis and fluorescence microscopy

Immunofluorescence analysis of transfected cells was performed 24 h after transfection [28]. After staining with Hoechst 33342 and MitoTracker Red, the cells were washed with DPBS, fixed in 4% paraformaldehyde in DPBS for 15 min, and permeabilized with 0.1% Triton X-100 in DPBS for 10 min at room temperature, followed by washing with 0.1% gelatin in DPBS. The permeabilized cells were incubated with anti-SAMM50 antibody (HPA034537) in DPBS for 1 h at room temperature. After washing with 0.1% gelatin in DPBS, the cells were incubated with anti-Rabbit IgG-FITC antibody for 1 h at room temperature. After washing with 0.1% gelatin in DPBS, the cells were observed using a Leica AF7000 fluorescence microscope (Leica, Solmser, Germany). Quantitative analysis of the mitochondrial localization of SAMM50 was performed by fluorescence microscopic observation of 50 immunofluorescence-positive (transfected) cells. The extent of mitochondrial localization was expressed as a percentage of the number of cells in which selective localization to mitochondria, localization to both mitochondria and cytoplasm, and selective localization to the cytoplasm was observed against the total number of transfected cells. Data are expressed as mean ± SD for 5 independent experiments.

### Immunoprecipitation

Samples were immunoprecipitated with a specific anti-SAMM50 antibody (HPA034537), as described previously [27].

### Statistic analysis

Statistical analysis was carried out using two-tailed *t* test (Microsoft Excel; Microsoft). The means of two distributions were considered significantly different if p < 0.05.

## Results

### Selection of candidate cDNA clones encoding human *N*-myristoylated proteins from a cDNA resource

**Fig 1:**
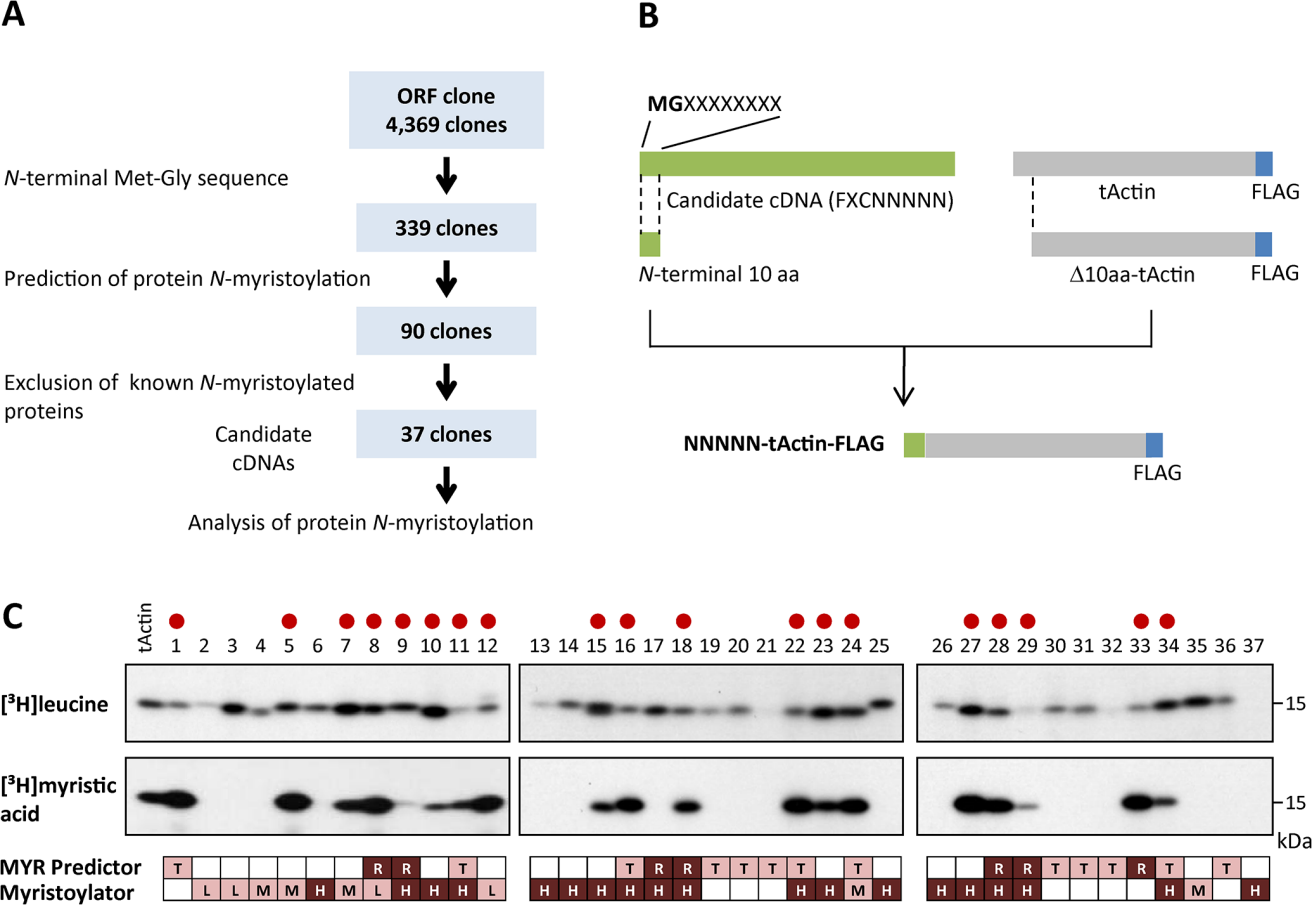
Selection of candidate cDNA clones encoding human N-myristoylated proteins from a cDNA resource. A. Strategy for selection of candidate cDNA clones encoding human *N*-myristoylated proteins from a cDNA resource. B. Schematic representation of the generation of NNNNN-tActin-FLAG with *N*-terminal 10 amino acid sequences of the ORFs of KOP cDNA clones at its *N*-terminus. C. Detection of protein *N*-myristoylation of NNNNN-tActin-FLAGs by metabolic labeling in an insect cell-free protein synthesis system. NNNNN-tActin-FLAGs were synthesized in the presence of [^3^H]leucine or [^3^H]myristic acid, and the labeled translation products were analyzed by SDS-PAGE and fluorography. The myristoylated samples are indicated by red circles. The results of the predictions of protein *N-*myristoylation using two prediction programs, The MYR Predictor and Myristoylator, are shown in the lower panels. R, T, and blank represent ‘Reliable’, ‘Twilight zone,’ and ‘No’ prediction from The MYR Predictor, respectively. H, M, L, and blank represent ‘High confidence’, ‘Medium confidence’, ‘Low confidence,’ and ‘No’ predictions from Myristoylator, respectively.

In order to search for human *N*-myristoylated proteins, 339 cDNA clones with *N*-terminal Met-Gly motifs were extracted from 4,369 KOP (Kazusa ORFeome project) human cDNA clones (FXC01948 ~ FXC23818) (Fig 1A). After applying the *N*-terminal sequence of the products of these 339 cDNA clones to two protein *N*-myristoylation prediction programs, The MYR Predictor and Myristoylator, 90 positively selected cDNA clones were indentified (S3 Table). From these cDNA clones, 53 clones coding for known *N*-myristoylated proteins identified by database searches were removed, and 37 cDNA clones were selected as potential candidates for human *N*-myristoylated proteins (Fig 1A). The samples analyzed are listed in S4 Table.

The susceptibility of human cDNA clones to protein *N-*myristoylation was evaluated using fusion proteins in which the *N*-terminal 10 amino acid residues were fused to an epitope-tagged model protein, tActin (Fig 1B). In this experiment, the fusion proteins were synthesized using an insect cell-free protein synthesis system in the presence of [^3^H]leucine or [^3^H]myristic acid, and then analyzed by SDS-PAGE and fluorography. As shown in the upper panels of Fig 1C, most of the cDNA clones were expressed, as determined by the incorporation of [^3^H]leucine. In contrast, the incorporation of [^3^H]myristic acid was observed for 19 out of 37 clones, as shown in the middle panels of Fig 1C. The results of the prediction for protein *N-*myristoylation using two prediction programs, The MYR Predictor and Myristoylator, are shown in the lower panels of Fig 1C.

### Analyses of protein *N-*myristoylation occurring in *in vitro* synthesized full-length cDNA products

**Fig 2:**
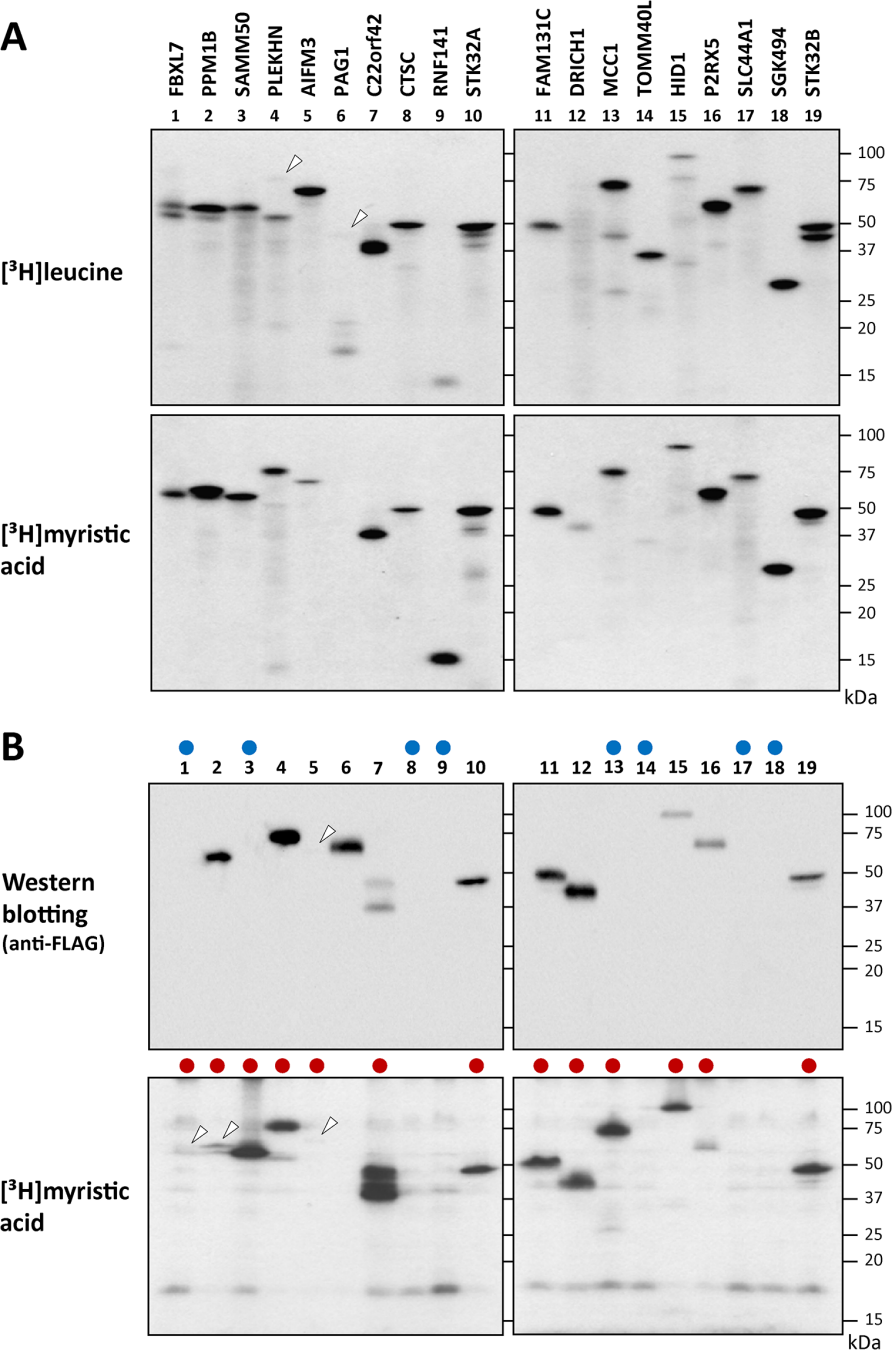
Detection of protein *N*-myristoylation of the gene products of 19 full-length cDNAs by metabolic labeling in an insect cell-free protein synthesis system and in transfected HEK293T cells. A. The gene products of 19 full-length cDNAs, in which efficient incorporation of [^3^H]myristic acid was observed with the tActin fusion proteins, were synthesized using an insect cell-free protein synthesis system in the presence of [^3^H]leucine or [^3^H]myristic acid. The labeled translation products were analyzed by SDS-PAGE and fluorography. Faint bands are indicated by arrowheads. B. The 19 full-length cDNAs analyzed in Fig 2A were transfected into HEK293T cells, and metabolic labeling with [^3^H]myristic acid was performed. The labeled translation products were separated by SDS-PAGE and then analyzed by western blotting using an anti-FLAG antibody or fluorography. The samples that showed no protein expression are indicated by blue circles in the upper panels. The samples in which protein *N*-myristoylation was observed are indicated by red boxes in the lower panels. Faint bands are indicated by arrowheads.

To determine whether the experimental results obtained with the tActin fusion proteins were consistent with those with the full-length cDNA products, metabolic labeling experiments were performed using the 19 full-length cDNAs in which incorporation of [^3^H]myristic acid was observed with the tActin fusion proteins. The samples analyzed are listed in S5 Table. As shown in the upper panels of Fig 2A, all the cDNA clones were expressed, as determined by the incorporation of [^3^H]leucine. In addition to the protein band with an expected molecular mass, some protein bands with lower molecular masses were observed with several cDNA clones, such as FBXL7 (lane 1), PLEKHN (lane 4), PAG1 (lane 6), MCC1 (lane 13), TOMM40L (lane 15), and STK32B (lane 19). The reason why this happened was not clear. The results of [^3^H]myristic acid incorporation revealed that the products of 18 out of 19 cDNA clones were *N-*myristoylated, as shown in the lower panels of Fig 2A. Protein *N*-myristoylation was not observed with the product of PAG1, as shown in lane 6 in the lower panel of Fig 2A.

### Analyses of protein *N-*myristoylation occurring in full-length cDNA products expressed in transfected human cells

**Fig 3:**
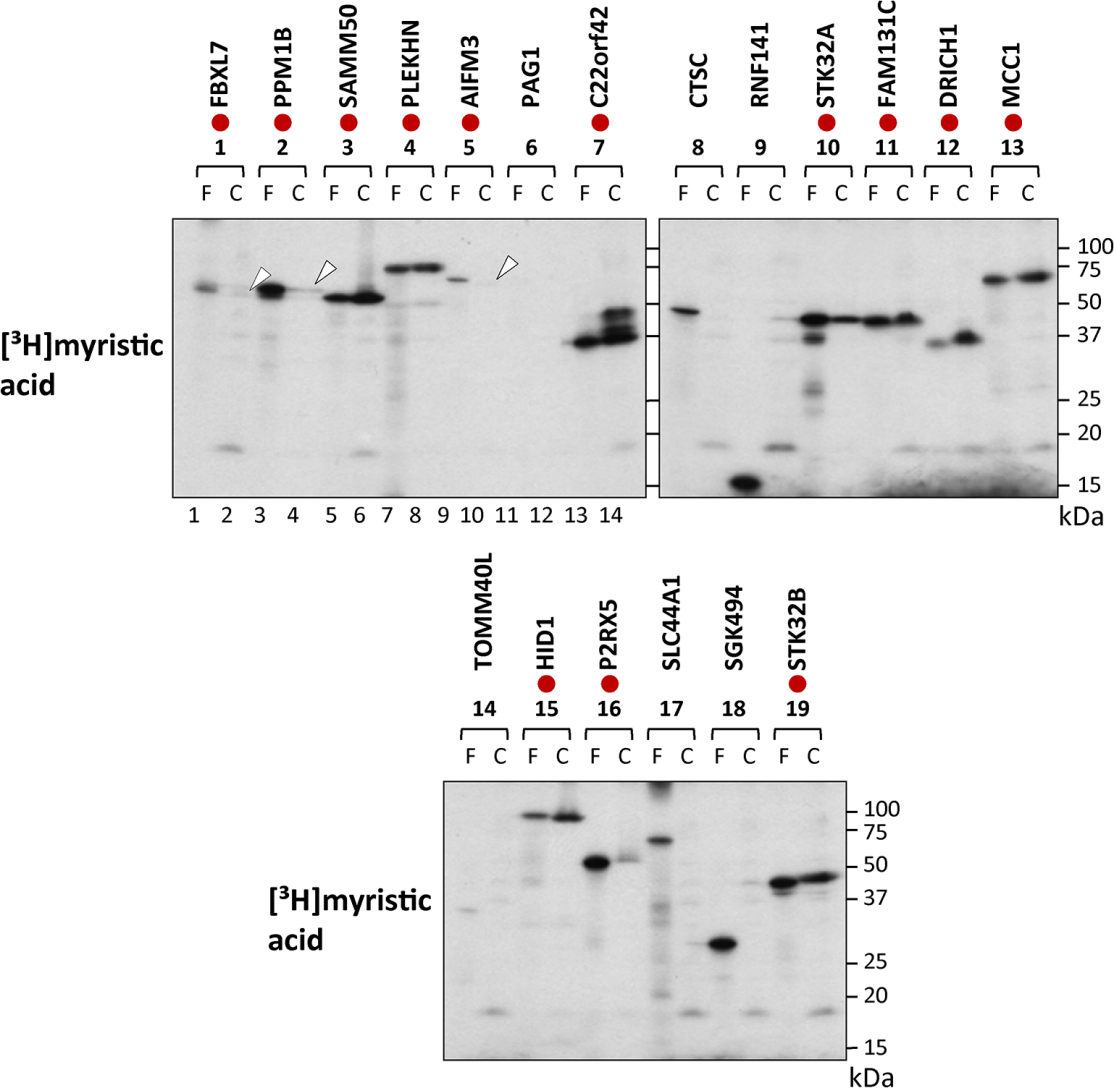
Comparison of the molecular size of [^3^H]myristic acid-labeled protein bands detected in transfected HEK293T cells with those detected in the insect cell-free protein synthesis system. The molecular sizes of [^3^H]myristic acid-labeled protein bands expressed in the two expression systems were compared with each other by SDS-PAGE analysis. The samples in which similar molecular sizes were observed are indicated by red circles. Faint bands are indicated by arrowheads. F; insect cell-free, C; HEK293T cells.

To determine whether results obtained by *in vitro* metabolic labeling in the insect cell-free protein synthesis system reflected the *in vivo* behavior of the cDNA products, metabolic labeling in transfected HEK293T (a human embryonic kidney cell line) cells was performed using the 19 full-length cDNAs analyzed in the insect cell-free protein synthesis system (S5 Table). In contrast to the insect cell-free protein synthesis system, not all the cDNA clones were expressed in transfected HEK293T cells. Protein synthesis was observed for 11 cDNA clones, as determined by the western blotting analysis using an anti-FLAG antibody. Protein expression was not observed with 8 cDNA clones, as indicated by blue circles in the upper panels of Fig 2B. As for protein *N-*myristoylation, obvious incorporation of [^3^H]myristic acid was observed for 13 out of 19 cDNA clones, as indicated by red circles in the lower panels of Fig 2B. Curiously, as shown in lanes 1, 3 and 13 in the lower panels of Fig 2B, [^3^H]myristic acid incorporation was observed with FBXL7, SAMM50 and MCC1, in which protein expression determined by western blotting analysis was not detected. These results suggested that the FLAG-tagged C-terminal region of these three proteins might be removed by proteolysis occurring in transfected HEK293T cells. To study this, the molecular sizes of [^3^H]myristic acid-labeled protein bands obtained in transfected HEK293T cells were compared with those obtained in the insect cell-free protein synthesis system by SDS-PAGE. As indicated by red circles in Fig 3, the molecular size of 13 [^3^H]myristic acid-labeled protein bands detected in transfected HEK293T cells were similar to those obtained using the cell-free protein synthesis system. For this experiment, overexposed fluorograms of the fluorography data of Fig 3 are shown in S1 Fig to demonstrate the presence of protein bands in lanes 2, 4 and 10. Thus, it seems likely that the FLAG-tagged C-terminal regions of FBXL7, SAMM50 and MCC1 were not removed in the transfected HEK293T cells. The reason why the expression of these three proteins could not be detected by western blotting analysis was not clear. It should be noted that [^3^H]myristic acid-labeled protein bands of C22orf42 detected in transfected cells showed several protein bands larger than that obtained using the cell-free protein synthesis system, as shown in lanes 13 and 14 in the upper left panel of Fig 3. In addition to protein *N*-myristoylation, other posttranslational modifications might occur with C22orf42 expressed in HEK293T cells. It was concluded from the experimental results described above that at least 13 out of 19 proteins tested were actually *N*-myristoylated.

### Characteristics of the gene products of 13 human cDNA clones found to be *N*-myristoylated in this study

The characteristics of the gene products of 13 human cDNA clones found to be *N-*myristoylated in this study are summarized in Table 1.

**Table 1:** The characteristics of the gene products of the 13 cDNA clones found to be *N-*myristoylated in this study. For prediction of protein *N-*myristoylation, the same abbreviations are used as in Fig 1C.

	FXC No.	Protein name	Gene name	Accession no.	Length (aa)	Protein function	Reference
1	FXC01999	F-box and leucine-rich repeat protein 7	FBXL7	AB463041	491	E3 ubiquitin ligase complex component	
2	FXC02617	Protein phosphatase 1B	PPM1B	AB464099	479	Protein phosphatase	[19,29]
3	FXC02844	Sorting and assembly machinery component 50 homolog	SAMM50	AB463278	469	Mitochondrial sorting and assembly machinery complex component	[20]
4	FXC02940	Pleckstrin homology domain containing, family N member 1	PLEKHN	AB463180	611	Unknown	[25]
5	FXC02961	Apoptosis-inducing factor, mitochondrion-associated, 3	AIFM3	AB462952	605	Apoptosis-inducing protein	
6	FXC03534	Uncharacterized protein C22orf42	C22orf42	AB527618	251	Unknown	
7	FXC03969	Serine/threonine-protein kinase 32A	STK32A	AB528042	396	Protein kinase	[25]
8	FXC04954	Family with sequence similarity 131, member C	FAM131C	AB463624	280	Unknown	
9	FXC05856	Aspartate-rich protein 1, chromosome 22 open reading frame 43	DRICH1	AB528170	228	Unknown	
10	FXC05945	Mutated in colorectal cancers	MCC1	AB527185	813	Cancer-related protein	
11	FXC10490	Protein HID1	HID1	AB527292	788	Cancer-related protein	[20,25,30]
12	FXC10528	Purinergic receptor P2X,	P2RX5	AB528606	422	Transmembrane receptor	[20]
13	FXC11252	Serine/threonine kinase 32B	STK32B	AB528925	414	Protein kinase	

Among these 13 proteins, 6 proteins (PPM1B, SAMM50, PLEKHN, STK32A, HID1, P2RX5) were recently reported to be *N*-myrsitoylated by cell-free and cellular metabolic labeling [25, 29] or by bioorthogonal reaction followed by MS-based identification [19, 20]. The analysis of the role of protein *N*-myristoylation on the intracellular localization or function has been performed on 4 proteins (PLEKHN, STK32A, PPM1B, HID1) [25, 29, 30] out of these 6 proteins.

The 13 *N*-myristoylated proteins found in this study included key components in various cellular signal transduction pathways such as a protein kinase, E3-ubiquitin ligase component, cancer-related protein, apoptosis-related protein, but also integral transmembrane proteins that play critical roles in cellular functions.

### Protein *N*-myristoylation strongly affects the mitochondrial targeting of SAMM50, an outer membrane protein of mitochondria

**Fig 4:**
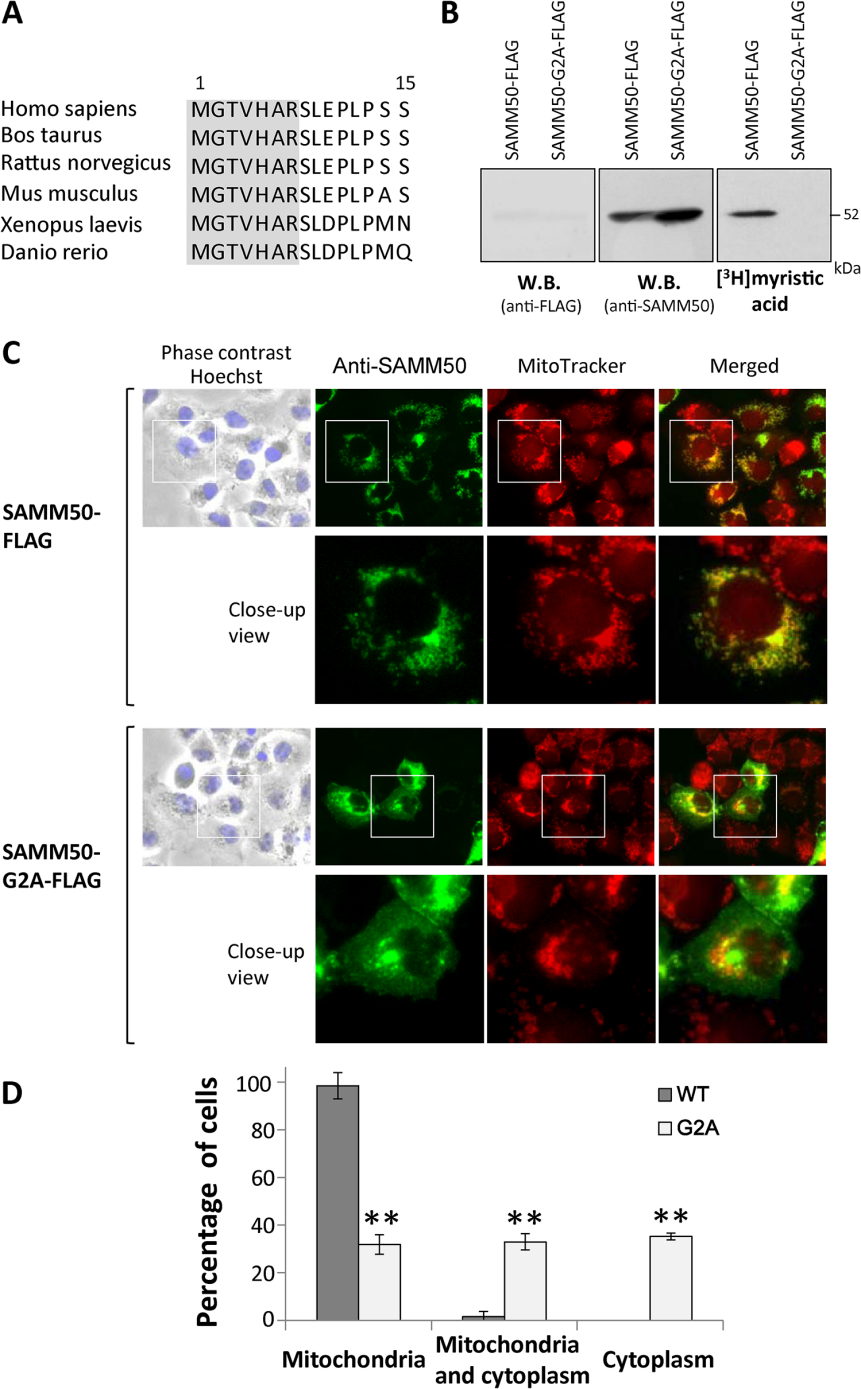
Protein *N*-myristoylation is required for proper targeting of SAMM50 to mitochondria. A. Interspecies alignment of the *N*-terminal sequences of SAMM50. *N*-myristoylation motifs are shown in grey in the *N*-terminal sequence. B. Detection of protein *N*-myristoylation of SAMM50 expressed in COS-1 cells. cDNAs encoding SAMM50-FLAG and SAMM50-G2A-FLAG were transfected into COS-1 cells, and their expression and the *N*-myristoylation of the products in total cell lysates were evaluated by western blotting analysis (using anti-FLAG or anti-SAMM50 antibodies) and [^3^H]myristic acid labeling, respectively. C. Intracellular localization of SAMM50-FLAG and SAMM50-G2A-FLAG was determined by immunofluorescence staining of COS-1 cells transfected with cDNAs encoding these two proteins using an anti-SAMM50 antibody. Mitochondria were detected using MitoTracker Red. Lower panels show a close-up image of the area outlined by a white box in the upper panels. D. Quantitative analysis of the intracellular localization of SAMM50-FLAG and SAMM50-G2A-FLAG. cDNAs encoding SAMM50-FLAG and SAMM50-G2A-FLAG were transfected into COS-1 cells and the intracellular localization of the expressed proteins in each cell was determined by immunofluorescence staining, and the extent of mitochondrial localization was compared. Quantitative analysis of the mitochondrial localization of SAMM50-FLAG and SAMM50-G2A-FLAG was performed by fluorescence microscopic observation of 50 immunofluorescence-positive (transfected) cells. The extent of mitochondrial localization was expressed as a percentage of the number of cells in which selective localization to mitochondria, localization to mitochondria and cytoplasm, and selective localization to cytoplasm was observed against the total number of transfected cells. Data are expressed as mean ± SD for five independent experiments. **P < 0.001 vs. wild-type.

In order to determine the physiological importance of protein *N*-myristoylation occurring on human *N*-myristoylated proteins found in this study, the role of protein *N*-myristoylation on the intracellular localization of SAMM50, an outer membrane protein of mitochondria, was investigated. SAMM50 is a central component of the sorting and assembly machinery (SAM) necessary for the assembly of β-barrel proteins in the mitochondrial outer membrane [31–33]. Interspecies alignments revealed that the *N*-terminal *N*-myristoylation motif were highly conserved among vertebrates, as shown in Fig 4A.

For this analysis, a non-*N*-myristoylated G2A mutant, in which Gly2 was replaced with Ala, was constructed and its intracellular localization was compared with that of wild-type protein. As shown in Fig 4B, efficient expression of wild-type and G2A mutant of SAMM50 tagged with FLAG-tag was observed as determined by western blotting using an anti-SAMM50 antibody. In contrast, protein expression was not detected by western blotting using an anti-FLAG antibody, as described previously. Metabolic labeling with [^3^H]myristic acid revealed that efficient incorporation of [^3^H]myristic acid was observed in wild-type SAMM50, but the incorporation was completely inhibited by replacing Gly2 with Ala. The results of immunofluorescence staining of COS-1 (simian virus 40-transformed African green monkey kidney cell line) cells transfected with these cDNAs revealed that protein *N*-myristoylation strongly affected the intracellular localization of SAMM50, as shown in Fig 4C. Immunofluorescence staining with the anti-SAMM50 antibody coupled with MitoTracker staining revealed that *N*-myristoylated SAMM50-FLAG exclusively localized to mitochondria, whereas the non-myristoylated G2A mutant localized mainly to the cytoplasm. In order to confirm the role of protein *N*-myristoylation in mitochondrial targeting of SAMM50, quantitative analysis of the intracellular localization of SAMM50-FLAG and SAMM50-G2A-FLAG expressed in COS-1 cells was performed by fluorescence microscopic observation. As a result, significant differences in intracellular localization were observed with these two proteins, as shown in Fig 4D. In the case of SAMM50-FLAG, most of the expressed proteins were specifically localized to the mitochondria. In contrast, as for SAMM50-G2A-FLAG, cytoplasmic localization (cytoplasm or mitochondria/cytoplasm) was observed in ~70% of transfected cells (Fig 4D). These results clearly indicated that protein *N*-myristoylation plays critical roles in the proper targeting of SAMM50 to mitochondria.

### Endogenous SAMM50 expressed in mammalian cells is *N*-myristoylated

**Fig 5:**
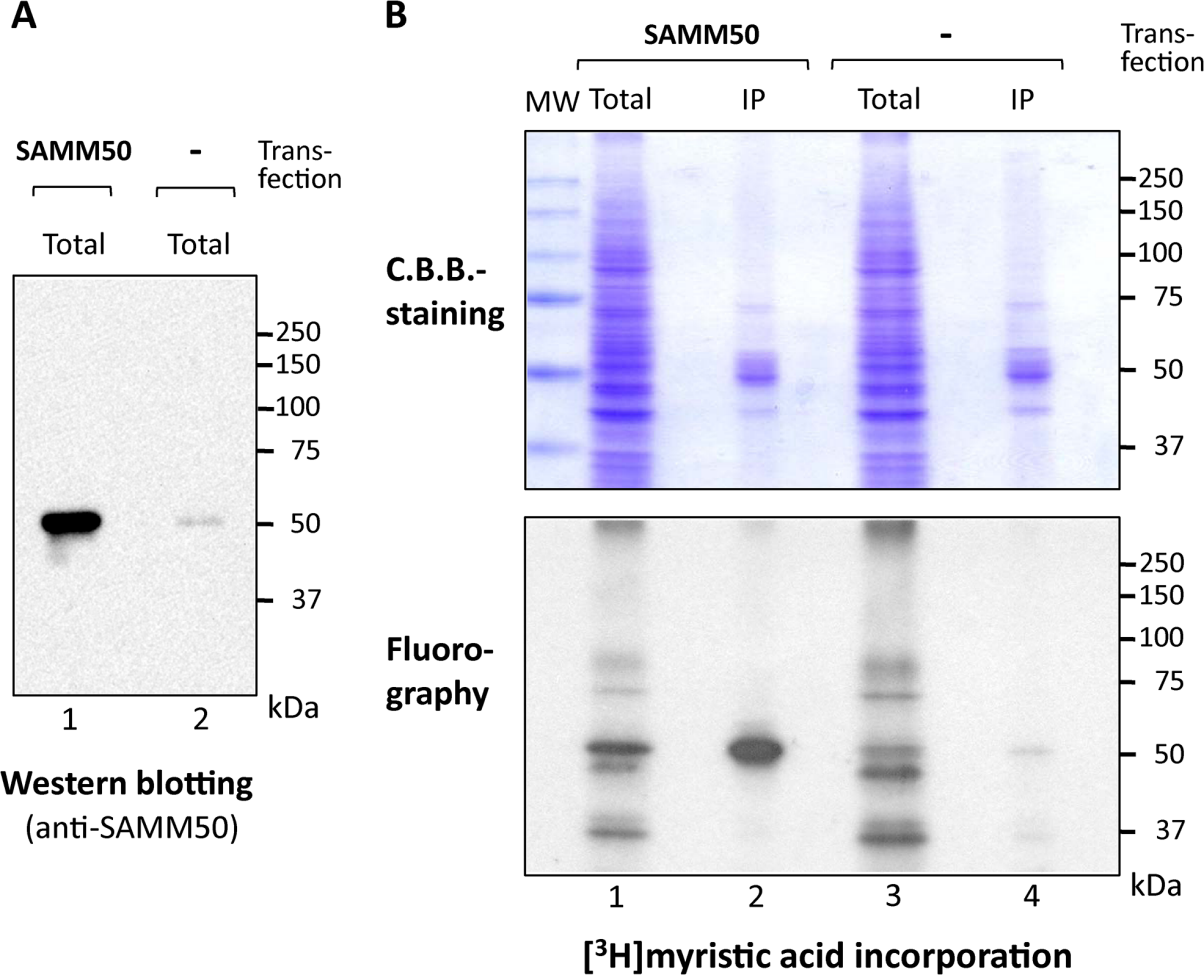
Endogenous SAMM50 expressed in COS-1 cells is *N*-myristoylated. Metabolic labeling of endogenous SAMM50 expressed in COS-1 cells with [^3^H]myristic acid was performed. Tag-free-SAMM50 exogenously expressed in COS-1 cells was used as a control. A. Expression of exogenously expressed Tag-free-SAMM50 and endogenous SAMM50 in COS-1 cells was determined by western blotting analysis using an anti-SAMM50 antibody. B. Protein *N*-myristoylation of exogenously expressed Tag-free-SAMM50 and endogenous SAMM50 in COS-1 cells was determined by [^3^H]myristic acid labeling. MW; molecular weight marker, IP; immunoprecipitated with anti-SAMM50 antibody. Upper and lower panels show the result of C.B.B.-staining and fluorography, respectively. Arrowheads in the upper panel indicate the position of heavy chain of IgG used for immunoprecipitation.

To determine whether protein *N*-myristoylation was observed on endogenous SAMM50 expressed in mammalian cells, metabolic labeling of endogenous SAMM50 expressed in COS-1 cells with [^3^H]myristic acid was performed. As shown in Fig 5A, lane 2, expression of endogenous SAMM50 was detected by western blotting analysis using an anti-SAMM50 antibody.

In this case, the molecular size of endogenous SAMM50 was similar to that of Tag-free-SAMM50 exogenously expressed in COS-1 cells (lane 1). As for [^3^H]myristic acid labeling, many labeled proteins were detected in the total cell lysates of COS-1 cells (B, lane 3 in lower panel). In samples immunoprecipitated with the anti-SAMM50 antibody, a [^3^H]myristic acid-labeled protein band of 52 kDa was detected (lane 4). The molecular size of this [^3^H]myristic acid-labeled protein band was similar to that observed with immunoprecipitated Tag-free-SAMM50 exogenously expressed in COS-1 cells (lane 2). These results clearly indicated that endogenous SAMM50 expressed in mammalian cells is *N*-myristoylated. When western blotting analysis was performed with 4 samples analyzed in Fig 5B, strong signals derived from heavy chain of IgG (~ 50 kDa) used for immunoprecipitation were detected on immunoprecipitated samples (S2 Fig). Therefore, the pattern of Coomassie brilliant blue (C.B.B.)-staining of the SDS-PAGE gel was shown in the upper panel of Fig 5B.

## Discussion

Protein *N*-myristoylation is one of the major forms of lipid modification that occurs in human cells. However, because of the lack of a simple and easy strategy to detect protein *N-*myristoylation, comprehensive identification of human *N*-myristoylated proteins has not been achieved. Recently, a novel approach based on chemical biology has become available for the study of protein *N*-myristoylation by taking advantage of bioorthogonal reactions [17–20]. For example, it was recently reported that metabolic labeling of cellular proteins with bioorthogonal myristic acid analogues and subsequent ligation with secondary reporters followed by enrichment and MS-based analysis successfully identified ~100 human *N*-myristoylated proteins expressed in HeLa cells [19]. Thus, this approach is extremely useful in detecting the *N*-myristoylated proteins expressed in intact cells. In this approach, relatively abundant constitutively expressed *N*-myristoylated proteins in the cells can be detected. However, for some cellular proteins, the amount of protein produced is lower than the detection limit of MS analysis. In addition, it is well known that only a limited sets of genes are expressed in particular cells. Hence, these approaches may not be suitable for the comprehensive identification of human *N*-myristoylated proteins expressed in whole human cells or tissues. Therefore, other approaches to identify human *N*-myristoylated proteins are required to fulfill the comprehensive identification of human *N*-myristoylated proteins.

It has been shown that protein *N*-myristoylation could be detected by metabolic labeling in various cell-free protein synthesis systems using cDNA clones [34–36]. In fact, we have demonstrated that a newly developed cell-free protein synthesis system (Transdirect insect cell) derived from insect cells [37] can be used for *in vitro* metabolic labeling assays, and that the metabolic labeling in this insect cell-free system is a simple and sensitive method to detect protein *N-*myristoylation [35]. In our previous study, to identify novel human *N-*myristoylated proteins, the susceptibility of human cDNA clones from human cDNA resource to protein *N-*myristoylation was evaluated by metabolic labeling and mass spectrometric analyses of proteins expressed using an insect cell-free protein synthesis system [21]. For this analysis, 141 cDNA clones with an *N*-terminal Met-Gly motif were selected as potential candidates from ~2,000 KOP (Kazusa ORFeome project) human cDNA clones. The susceptibility of these cDNA clones to protein *N-*myristoylation was first evaluated using fusion proteins, in which the *N*-terminal 10 amino acid residues were fused to an epitope-tagged model protein. Then, protein *N-*myristoylation on the gene product of the full-length cDNA was evaluated by metabolic labeling experiments both in an insect cell-free protein synthesis system and in transfected COS-1 cells. As a result, the products of 27 out of ~2,000 cDNA clones were found to be *N*-myristoylated. Among them, 18 proteins were novel *N-*myristoylated proteins that had not been reported previously. These results indicated that this strategy is useful for the identification of human *N-*myristoylated proteins from human cDNA resources. However, in this strategy, the efficiency of the detection of *N*-myristoylated protein from tested cDNA clones was low (27 out of 141 tested proteins). Therefore, in the present study, in order to increase the detection efficiency, cell-free- and cellular metabolic labeling experiments coupled with bioinformatic prediction systems for protein *N*-myristoylation were performed to identify physiologically important human *N*-myristoylated proteins.

A bioinformatic approach is a powerful strategy to perform comprehensive identification of *N-*myristoylated proteins [22, 23, 38, 39]. Two prediction programs, The MYR Predictor and Myristoylator are available as public WWW-servers [22, 23]. In the previous study, to evaluate the reliability of these prediction programs, experimental results of metabolic labeling with 141 proteins with a Met-Gly sequence at the *N*-terminus were compared with the results of the prediction obtained by the two prediction programs [21]. As a result, it was revealed that the reliability of The MYR Predictor was high; however, there were also a considerable number of false-negative predictions. In contrast, the Myristoylator predicted many more *N-*myristoylated proteins, but there were many false-positive predictions. When the positively predicted proteins by either of the two prediction programs were combined, most of the *N*-myristoylated proteins were predicted correctly. Therefore, in the present study, the proteins predicted to be *N*-myristoylated by either of the two prediction programs were used as candidates for human *N*-myristoylated proteins.

In order to identify physiologically important human *N*-myristoylated proteins, the susceptibility of the human cDNA clones in human cDNA resource to protein *N*-myristoylation was evaluated by metabolic labeling in a cell-free protein synthesis system coupled with bioinformatic prediction systems. For this analysis, 90 cDNA clones with an *N*-terminal Met-Gly motif predicted to be *N*-myristoylated by two bioinformatic *N*-myristoylation prediction systems were selected from ~4,400 Kazusa ORFeome project (KOP) human cDNA clones (FXC01948 ~ FXC23818) (S1 Table). After database searches for known *N*-myristoylated proteins, 37 cDNA clones were selected as potential candidates for human *N*-myristoylated proteins (S2 Table). The susceptibility of these cDNA clones to protein *N*-myristoylation was first evaluated using fusion proteins, in which the *N*-terminal 10 amino acid residues were fused to an epitope-tagged model protein (Fig 1). Then, protein *N*-myristoylation on the gene product of the full-length cDNA was evaluated by metabolic labeling experiments both in an insect cell-free protein synthesis system and in transfected human cells (Figs 2 and 3).

As a result, the products of 13 cDNA clones (FBXL7, PPM1B, SAMM50, PLEKHN, AIFM3, C22orf42, STK32A, FAM131C, DRICH1, MCC1, HID1, P2RX5, STK32B) were found to be human *N*-myristoylated proteins (Table 1). Among these 13 proteins, 6 proteins (PPM1B, SAMM50, PLEKHN, STK32A, HID1, P2RX5) were recently demonstrated to be *N*-myrsitoylated [19, 20, 25, 29], and the analysis of the role of protein *N*-myristoylation on the intracellular localization or function were reported on 4 proteins (PLEKHN, STK32A, PPM1B, HID1) [25, 29, 30]. These human *N*-myristoylated proteins contained not only physiologically important proteins such as a protein kinase, E3-ubiquitin ligase component, cancer-related protein, apoptosis-related protein, but also integral transmembrane proteins that play critical roles in cellular functions.

As for protein *N*-myristoylation occurring on integral membrane proteins, we have recently demonstrated that protein Lunapark, a double-spanning integral membrane protein of the ER, is *N*-myristoylated and the *N-*myristoylation of protein Lunapark strongly affected ER morphological changes induced by this protein [40]. Thus, it seems likely that protein *N*-myristoylation plays critical role in the function of integral membrane proteins. However, so far, very few integral membrane proteins has been found to be *N*-myristoylated. SAMM50, one of the human *N*-myristoylated proteins found in this study, is an integral membrane protein of the outer membrane of mitochondria. Therefore, we focused our attention on the protein *N*-myristoylation occurring on SAMM50. Analysis of the role of protein *N*-myristoylation on the intracellular localization of SAMM50 by immunofluorescence analysis revealed that protein *N*-myristoylation strongly affected the subcellular localization of SAMM50. *N*-myristoylated SAMM50-FLAG was found to localize exclusively to mitochondria, whereas a non-myristoylated G2A mutant localized mainly to the cytoplasm (Fig 4C and 4D). Furthermore, immunoprecipitiation analysis of [^3^H]myristic acid-labeled cellular proteins revealed that endogenous SAMM50 is *N*-myristoylated (Fig 5). Thus, it was demonstrated that SAMM50 expressed in intact cells is indeed *N*-myristoylated, and this modification plays a critical role in the intracellular localization of this protein. In eukaryotic cellular proteins, only very few integral membrane proteins have been demonstrated to be *N*-myristoylated. One well-characterized example of an *N*-myristoylated integral membrane protein in eukaryotes is mammalian NADH-cytochrome b(5) reductase (b5R). This protein is a single-spanning membrane protein with N-exo/C-cyto orientation, and it is dually targeted to the ER and mitochondrial outer membranes [41, 42]. In b5R, protein *N*-myristoylation is required for targeting to the mitochondria, with a non-myristoylated mutant exclusively localized to the ER [43]. It was further revealed that protein *N*-myristoylation interferes with the interaction of the nascent chain with the signal recognition particle, so that a portion of the nascent chains escape from cotranslational integration into the ER and can be post-translationally targeted to the mitochondrial outer membrane [44]. SAMM50 is a β-barrel protein that resides within the outer membrane of mitochondria [32, 33]. Unlike most of the transmembrane proteins that are anchored in the lipid bilayer via membrane spanning α-helices, β-barrel proteins transverse the membrane by interconnected β-strands [45]. It was revealed that all the mitochondrial outer membrane β-barrel proteins carry internal non-cleavable targeting and sorting signals [46]. Because SAMM50 does not contain a hydrophobic sequence that interacts with the signal recognition particle, it is probable that protein *N*-myristoylation of SAMM50 does not affect the interaction of the nascent polypeptide with the signal recognition particle. In fact, most of the non-myristoylated G2A-mutant of SAMM50 did not localize to ER but was found to localize to the cytoplasm (Fig 4C). The molecular mechanism by which protein *N*-myristoylation affects the mitochondrial targeting of SAMM50 is not clear. β-barrel proteins do not enter the outer mitochondrial membrane from the cytosolic side but rather are transferred via the TOM complex into the intermembrane space and are subsequently directed to the outer membrane [32, 33]. Therefore, protein *N*-myristoylation might positively affect these transport mechanisms of β-barrel proteins. Further studies are required to clarify the role of protein *N*-myristoylation on the targeting and integration of SAMM50 into the outer membrane of mitochondria. TOMM40L, an another outer membrane protein of mitochondria, is included in 18 proteins in which the product of full-length cDNA was found to be *N*-myristoylated in the insect cell-free protein synthesis system in this study. In addition, it was recently reported that TOMM40L is *N*-myristoylated in intact human cells [19, 20]. Thus, it is possible that protein *N*-myristoylation positively affect the mitochondrial targeting of TOMM40L.

In the present study, in order to identify physiologically important human *N*-myristoylated proteins from cDNA resources, cell-free- and cellular metabolic labeling experiments coupled with bioinformatic prediction systems for protein *N*-myristoylation were performed using KOP human cDNA clones as a model cDNA clones. As a result, the products of 13 cDNA clones including many physiologically important proteins were found to be human *N*-myristoylated proteins. In this case, however, it should be noted that this method is useful to screen human *N-*myristoylated proteins, but the actual modification status and the role of the protein *N*-myristoylation on the protein function should be confirmed or studied in the intact human cells by the biochemical analyses such as those performed on SAMM50 in this study. Four proteins out of 13 *N*-myristoylated proteins found in this study are functionally unknown proteins. Since many of the *N*-myristoylated proteins play critical roles in cellular function, the finding of protein *N*-myristoylation may aid in the future functional characterization of these proteins.

The number of human proteins with an *N*-terminal Met-Gly sequence in all the human proteins listed in the Swiss-Prot protein knowledgebase (~46,000 proteins including isoforms) is approximately 3,700. Thus, it is expected that many physiologically important *N*-myristoylated proteins will be identified when the same approach is performed on larger cDNA resources that include many human cDNAs.

## Supporting Information

S1 Fig Comparison of the molecular size of [^3^H]myristic acid-labeled protein bands detected in transfected HEK293T cells with those detected in the insect cell-free protein synthesis system. The overexposed fluorograms of the fluorography data of Fig 3 are shown to demonstrate the presence of protein bands in lanes 2, 4 and 10.
(TIF)

S2 Fig The result of western blotting analysis of 4 samples analyzed in Fig 5B is shown. Arrowheads indicate the position of heavy chain of IgG (~ 50kDa) used for immunoprecipitation.
(TIF)

S1 Table The nucleotide sequences of oligonucleotides used in this study. (DOC)

S2 Table The strategies for construction of pTD1 or pcDNA3 plasmids including full-length KOP cDNA clones. (DOC)

S3 Table The results of the prediction for protein *N*-myristoylation of 90 cDNA clones using two prediction programs, The MYR Predictor and Myristoylator. (DOCX)

S4 Table The list of cDNA samples analyzed in Fig 1. (DOCX)

S5 Table The list of cDNA samples analyzed in Figs 2 and 3. (DOCX)
