# Supplementary figures and images for: Identification of Human N-Myristoylated Proteins from Human Complementary DNA Resources by Cell-Free and Cellular Metabolic Labeling Analyses
Source: PLoS One. 2015 Aug 26;10(8):e0136360. doi: 10.1371/journal.pone.0136360 (PMC4550359; doi:10.1371/journal.pone.0136360)

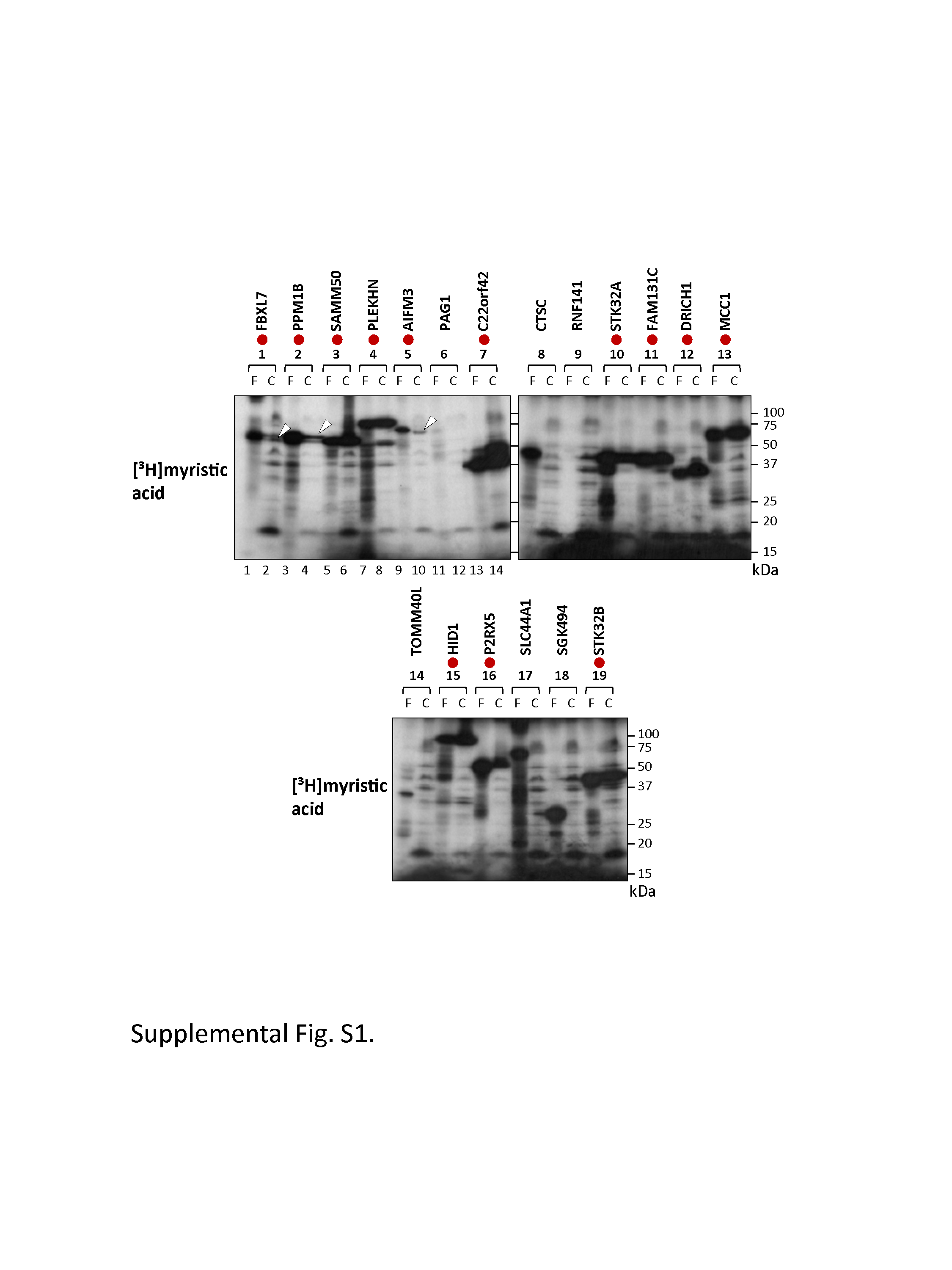

Supplement: S1 Fig — The overexposed fluorograms of the fluorography data of Fig 3 are shown to demonstrate the presence of protein bands in lanes 2, 4 and 10. (TIF) [file pone.0136360.s001.tif]

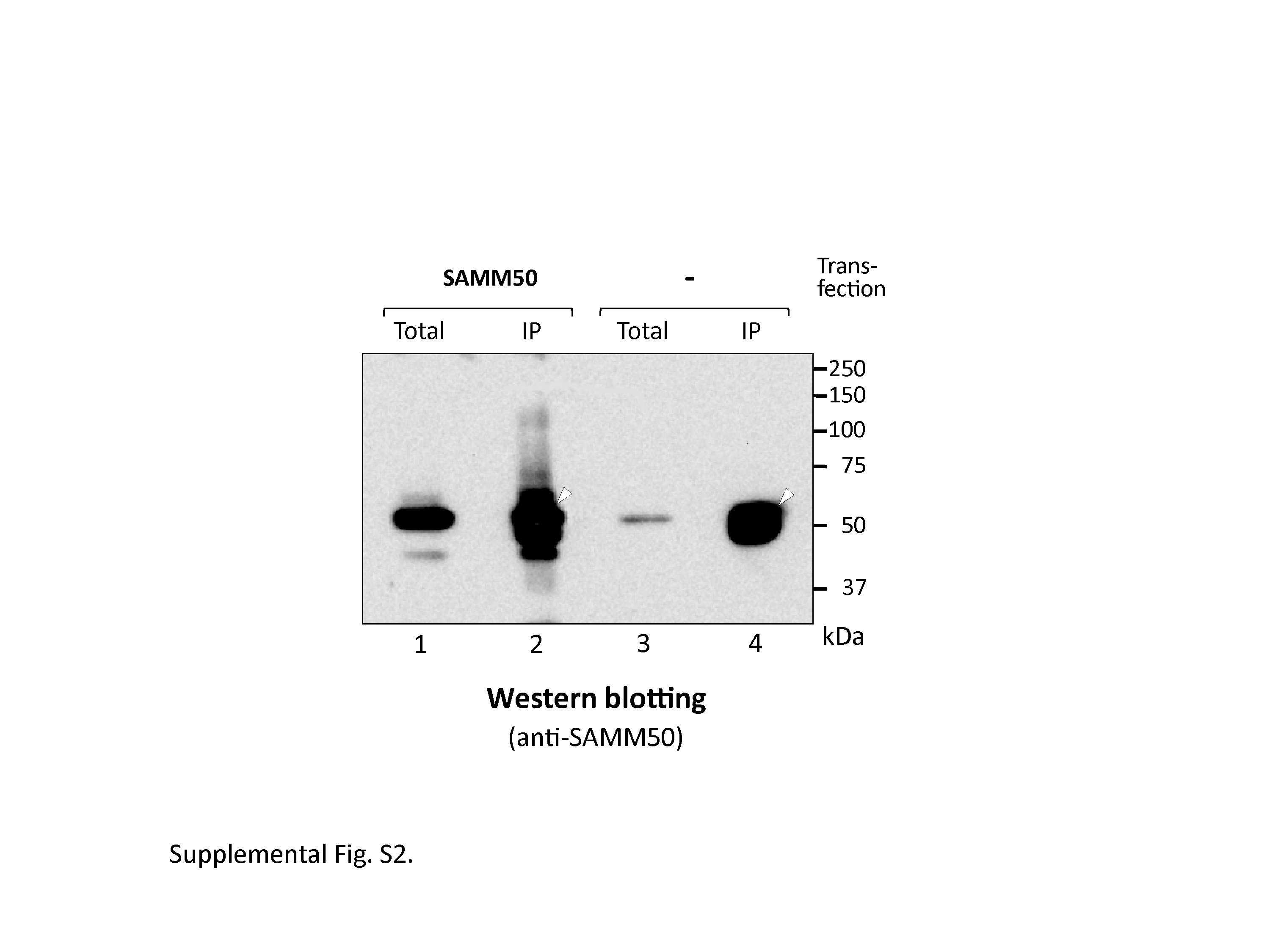

Supplement: S2 Fig — Arrowheads indicate the position of heavy chain of IgG (~ 50kDa) used for immunoprecipitation. (TIF) [file pone.0136360.s002.tif]
