# Supplementary material for: Identification of Human N-Myristoylated Proteins from Human Complementary DNA Resources by Cell-Free and Cellular Metabolic Labeling Analyses
Source: PLoS One. 2015 Aug 26;10(8):e0136360. doi: 10.1371/journal.pone.0136360 (PMC4550359; doi:10.1371/journal.pone.0136360)
Supplement: S1 Table — (DOC) [file pone.0136360.s003.doc]

**Supplemental Table S1. (1)**

The nucleotide sequences of oligonucleotides used in this study

| Primer | Primer | Sequence |
| --- | --- | --- |
| Primer-N1 | EcoRI-tAct11-N | 5’-ATATGAATTCCGGTTCCGCTGCCCT-3’ |
| Primer-C1 | XbaI-stop-Flag-C | GCGCTCTAGATTACTTGTCATCGTC |
| KOP01999 | EcoRV-FXC01999 10aa-EcoRI-N | ATCATGGGCGCGAACAATGGCAAACAGTACGGCG |
| KOP01999R | EcoRI-FXC01999 10aa-EcoRV-C | AATTCGCCGTACTGTTTGCCATTGTTCGCGCCCATGAT |
| KOP02047 | EcoRV-FXC02047 10aa-EcoRI-N | ATCATGGGGGACTCAGGATCAAGACGATCTACCG |
| KOP02047R | EcoRI-FXC02047 10aa-EcoRV-C | AATTCGGTAGATCGTCTTGATCCTGAGTCCCCCATGAT |
| KOP02078 | EcoRV-FXC02078 10aa-EcoRI-N | ATCATGGGAGTGGACATCCGCCATAACAAGGACG |
| KOP02078R | EcoRI-FXC02078 10aa-EcoRV-C | AATTCGTCCTTGTTATGGCGGATGTCCACTCCCATGAT |
| KOP02452 | EcoRV-FXC02452 10aa-EcoRI-N | ATCATGGGCAGCCCCTGGAACGGCAGCGACGGCG |
| KOP02452R | EcoRI-FXC02452 10aa-EcoRV-C | AATTCGCCGTCGCTGCCGTTCCAGGGGCTGCCCATGAT |
| KOP02617 | EcoRV-FXC02617 10aa-EcoRI-N | ATCATGGGTGCATTTTTGGATAAACCCAAAACTG |
| KOP02617R | EcoRI-FXC02617 10aa-EcoRV-C | AATTCAGTTTTGGGTTTATCCAAAAATGCACCCATGAT |
| KOP02819 | EcoRV-FXC02819 10aa-EcoRI-N | ATCATGGGAAGAATGTCTTCCAAGCAAGCCACCG |
| KOP02819R | EcoRI-FXC02819 10aa-EcoRV-C | AATTCGGTGGCTTGCTTGGAAGACATTCTTCCCATGAT |
| KOP02844 | EcoRV-FXC02844 10aa-EcoRI-N | ATCATGGGGACTGTGCACGCCCGGAGTTTGGAGG |
| KOP02844R | EcoRI-FXC02844 10aa-EcoRV-C | AATTCCTCCAAACTCCGGGCGTGCACAGTCCCCATGAT |
| KOP02940 | EcoRV-FXC02940 10aa-EcoRI-N | ATCATGGGGAACAGCCACTGTGTCCCTCAGGCCG |
| KOP02940R | EcoRI-FXC02940 10aa-EcoRV-C | AATTCGGCCTGAGGGACACAGTGGCTGTTCCCCATGAT |
| KOP02961 | EcoRV-FXC02961 10aa-EcoRI-N | ATCATGGGCGGCTGCTTCTCCAAACCCAAACCAG |
| KOP02961R | EcoRI-FXC02961 10aa-EcoRV-C | AATTCTGGTTTGGGTTTGGAGAAGCAGCCGCCCATGAT |
| KOP03470 | EcoRV-FXC03470 10aa-EcoRI-N | ATCATGGGGCCCGCGGGGAGCCTGCTGGGCAGCG |
| KOP03470R | EcoRI-FXC03470 10aa-EcoRV-C | AATTCGCTGCCCAGCAGGCTCCCCGCGGGCCCCATGAT |
| KOP03534 | EcoRV-FXC03534 10aa-EcoRI-N | ATCATGGGGAGCAAACTGACTTGCTGCCTGGGCG |
| KOP03534R | EcoRI-FXC03534 10aa-EcoRV-C | AATTCGCCCAGGCAGCAAGTCAGTTTGCTCCCCATGAT |
| KOP03565 | EcoRV-FXC03565 10aa-EcoRI-N | ATCATGGGTGCTGGGCCCTCCTTGCTGCTCGCCG |
| KOP03565R | EcoRI-FXC03565 10aa-EcoRV-C | AATTCGGCGAGCAGCAAGGAGGGCCCAGCACCCATGAT |
| KOP03640 | EcoRV-FXC03640 10aa-EcoRI-N | ATCATGGGCAGCGACCCGAGCGCGCCCGGACGGG |
| KOP03640 | EcoRI-FXC03640 10aa-EcoRV-C | AATTCCCGTCCGGGCGCGCTCGGGTCGCTGCCCATGAT |
| KOP03765 | EcoRV-FXC03765 10aa-EcoRI-N | ATCATGGGAGACAAGAAGAGCCCCACCAGGCCGG |
| KOP03765R | EcoRI-FXC03765 10aa-EcoRV-C | AATTCCGGCCTGGTGGGGCTCTTCTTGTCTCCCATGAT |
| KOP03868 | EcoRV-FXC03868 10aa-EcoRI-N | ATCATGGGACAGCAAATTTCGGATCAGACACAGG |
| KOP03868R | EcoRI-FXC03868 10aa-EcoRV-C | AATTCCTGTGTCTGATCCGAAATTTGCTGTCCCATGAT |
| KOP03969 | EcoRV-FXC03969 10aa-EcoRI-N | ATCATGGGAGCGAACACTTCAAGAAAACCACCAG |
| KOP03969R | EcoRI-FXC03969 10aa-EcoRV-C | AATTCTGGTGGTTTTCTTGAAGTGTTCGCTCCCATGAT |
| KOP04325 | EcoRV-FXC04325 10aa-EcoRI-N | ATCATGGGCAATTTACTTAGTAAATTTAGACCCG |
| KOP04325R | EcoRI-FXC04325 10aa-EcoRV-C | AATTCGGGTCTAAATTTACTAAGTAAATTGCCCATGAT |
| KOP04954 | EcoRV-FXC04954 10aa-EcoRI-N | ATCATGGGCTCCTGCGTGTCGCGAGACCTGTTCG |

**Supplemental Table S1. (2)**

The nucleotide sequences of oligonucleotides used in this study

| Primer | Primer | Sequence |
| --- | --- | --- |
| KOP04954R | EcoRI-FXC04954 10aa-EcoRV-C | AATTCGAACAGGTCTCGCGACACGCAGGAGCCCATGAT |
| KOP05791 | EcoRV-FXC05791 10aa-EcoRI-N | ATCATGGGCTGCTGTGGCTGCTCCAGAGGCTGTG |
| KOP05791R | EcoRI-FXC05791 10aa-EcoRV-C | AATTCACAGCCTCTGGAGCAGCCACAGCAGCCCATGAT |
| KOP05792 | EcoRV-FXC05792 10aa-EcoRI-N | ATCATGGGCTGCTCTGGCTGCTCTGGAGGCTGTG |
| KOP05792R | EcoRI-FXC05792 10aa-EcoRV-C | AATTCACAGCCTCCAGAGCAGCCAGAGCAGCCCATGAT |
| KOP05856 | EcoRV-FXC05856 10aa-EcoRI-N | ATCATGGGGAATATACTGACCTGTTGTATCAACG |
| KOP05856R | EcoRI-FXC05856 10aa-EcoRV-C | AATTCGTTGATACAACAGGTCAGTATATTCCCCATGAT |
| KOP05945 | EcoRV-FXC05945 10aa-EcoRI-N | ATCATGGGTTTGCTCCATTCAGCAGCCCTGGCAG |
| KOP05945R | EcoRI-FXC05945 10aa-EcoRV-C | AATTCTGCCAGGGCTGCTGAATGGAGCAAACCCATGAT |
| KOP07187 | EcoRV-FXC07187 10aa-EcoRI-N | ATCATGGGGAACACATTGGGCCTGGCACCAATGG |
| KOP07187R | EcoRI-FXC07187 10aa-EcoRV-C | AATTCCATTGGTGCCAGGCCCAATGTGTTCCCCATGAT |
| KOP07438 | EcoRV-FXC07438 10aa-EcoRI-N | ATCATGGGGAAAAAACAAAACAAGAAGAAAGTGG |
| KOP07438R | EcoRI-FXC07438 10aa-EcoRV-C | AATTCCACTTTCTTCTTGTTTTGTTTTTTCCCCATGAT |
| KOP07771 | EcoRV-FXC07771 10aa-EcoRI-N | ATCATGGGAGAAGAAGCATCCATTGTGTCTTCAG |
| KOP07771R | EcoRI-FXC07771 10aa-EcoRV-C | AATTCTGAAGACACAATGGATGCTTCTTCTCCCATGAT |
| KOP10490 | EcoRV-FXC10490 10aa-EcoRI-N | ATCATGGGGTCGACCGACTCCAAGCTGAACTTCG |
| KOP10490R | EcoRI-FXC10490 10aa-EcoRV-C | AATTCGAAGTTCAGCTTGGAGTCGGTCGACCCCATGAT |
| KOP10528 | EcoRV-FXC10528 10aa-EcoRI-N | ATCATGGGGCAGGCGGGCTGCAAGGGGCTCTGCG |
| KOP10528R | EcoRI-FXC10528 10aa-EcoRV-C | AATTCGCAGAGCCCCTTGCAGCCCGCCTGCCCCATGAT |
| KOP10683 | EcoRV-FXC10683 10aa-EcoRI-N | ATCATGGGCTGCTGCAGCTCCGCCTCCTCCGCCG |
| KOP10683R | EcoRI-FXC10683 10aa-EcoRV-C | AATTCGGCGGAGGAGGCGGAGCTGCAGCAGCCCATGAT |
| KOP10889 | EcoRV-FXC10889 10aa-EcoRI-N | ATCATGGGCAGTGTGCGAACCAACCGCTACAGCG |
| KOP10889R | EcoRI-FXC10889 10aa-EcoRV-C | AATTCGCTGTAGCGGTTGGTTCGCACACTGCCCATGAT |
| KOP11186 | EcoRV-FXC11186 10aa-EcoRI-N | ATCATGGGCTGCTGTGGCTGTTCCGAAGGCTGTG |
| KOP11186R | EcoRI-FXC11186 10aa-EcoRV-C | AATTCACAGCCTTCGGAACAGCCACAGCAGCCCATGAT |
| KOP11187 | EcoRV-FXC11187 10aa-EcoRI-N | ATCATGGGCTGTTGCGGCTGCTCCGGAGGCTGTG |
| KOP11187 | EcoRI-FXC11187 10aa-EcoRV-C | AATTCACAGCCTCCGGAGCAGCCGCAACAGCCCATGAT |
| KOP11232 | EcoRV-FXC11232 10aa-EcoRI-N | ATCATGGGAGCAGTAAGCTGTCGGCAGGGGCAGG |
| KOP11232R | EcoRI-FXC11232 10aa-EcoRV-C | AATTCCTGCCCCTGCCGACAGCTTACTGCTCCCATGAT |
| KOP11252 | EcoRV-FXC11252 10aa-EcoRI-N | ATCATGGGCGGGAACCACTCCCACAAGCCCCCCG |
| KOP11252R | EcoRI-FXC11252 10aa-EcoRV-C | AATTCGGGGGGCTTGTGGGAGTGGTTCCCGCCCATGAT |
| KOP11288 | EcoRV-FXC11288 10aa-EcoRI-N | ATCATGGGTGACAAGGGAACAGGCAACCATTCAG |
| KOP11288R | EcoRI-FXC11288 10aa-EcoRV-C | AATTCTGAATGGTTGCCTGTTCCCTTGTCACCCATGAT |
| KOP11366 | EcoRV-FXC11366 10aa-EcoRI-N | ATCATGGGCTGCAGAAGAACTAGAGAAGGACCAG |
| KOP11366R | EcoRI-FXC11366 10aa-EcoRV-C | AATTCTGGTCCTTCTCTAGTTCTTCTGCAGCCCATGAT |
| KOP11932 | EcoRV-FXC11932 10aa-EcoRI-N | ATCATGGGAGACCCGGGGTCGGAAATAATAGAAG |
| KOP11932R | EcoRI-FXC11932 10aa-EcoRV-C | AATTCTTCTATTATTTCCGACCCCGGGTCTCCCATGAT |

**Supplemental Table S1. (3)**

The nucleotide sequences of oligonucleotides used in this study

| Primer | Primer | Sequence |
| --- | --- | --- |
| Primer-N2 | EcoRI-FXC1999-N | gcatgaattcatgggcgcgaacaatg |
| Primer-C2 | FXC1999-Flag-XbaI-C | gcgctctagattacttgtcatcgtcatccttgtagtcgaagaaagccgggtt |
| Primer-N3 | EcoRV- FXC02617-N | gcatgatatcatgggtgcatttttg |
| Primer-C3 | FXC02617-Flag-EcoRI-C | gcgcgaattcttacttgtcatcgtcatccttgtagtctattttttcaccact |
| Primer-N4 | BamHI-FXC2844-N | atatggatccatggggactgtgcac |
| Primer-C4 | FXC2844-Flag-XbaI-C | gcgctctagattacttgtcatcgtcatccttgtagtccaggaaccttatccc |
| Primer-N5 | EcoRV- FXC02940-N | gatagatatcatggggaacagccac |
| Primer-C5 | FXC02940-Flag-EcoRI-C | gcgcgaattcttacttgtcatcgtcatccttgtagtcgatccactgcacaag |
| Primer-N6 | EcoRI-FXC03470-N | atatgaattcatggggcccgcgggg |
| Primer-C6 | FXC03470-Flag-stop-XbaI-C | gcgctctagattacttgtcatcgtcatccttgtagtcgagcctggtaatatc |
| Primer-N7 | EcoRV-FXC03534-N | gtacgatatcatggggagcaaactg |
| Primer-C7 | FXC03534-Flag-stop-EcoRI | aggggaattcttacttgtcatcgtcatccttgtagtcaagccggagaagtcc |
| Primer-N8 | EcoRV-FXC3565-N | atatgatatcatgggtgctgggccc |
| Primer-C8 | FXC3565-Flag-XbaI-C | gcgctctagattacttgtcatcgtcatccttgtagtccaatttaggaattgg |
| Primer-N9 | EcoRI-FXC03868-N | gcgcgaattcatgggacagcaaatt |
| Primer-C9 | FXC03868-Flag-XbaI-C | atgctctagattacttgtcatcgtcatccttgtagtctggcctgtggggctg |
| Primer-N10 | EcoRV- FXC03969-N | gatagatatcatgggagcgaacact |
| Primer-C10 | FXC03969- Flag-EcoRI-C | gcgcgaattcttacttgtcatcgtcatccttgtagtccaagttgttattctg |
| Primer-N11 | EcoRI-FXC04954-N | atatgaattcatgggctcctgcgtg |
| Primer-C11 | FXC04954-Flag-stop-XbaI-C | gcgctctagattacttgtcatcgtcatccttgtagtcgttatagaacacctc |
| Primer-N12 | EcoRI-FXC05856-N | gcgcgaattcatggggaatatactg |
| Primer-C12 | FXC05856-Flag-stop-XbaI-C | gcgctctagattacttgtcatcgtcatccttgtagtcccctggatgaatctc |
| Primer-N13 | EcoRV- FXC05945-N | gcatgatatcatgggtttgctccat |
| Primer-C13 | FXC05945- Flag-EcoRI-C | gcgcgaattcttacttgtcatcgtcatccttgtagtcaagcgaagtttcatt |
| Primer-N14 | EcoRI- FXC07187-N | gcgcgaattcatggggaacacattg |
| Primer-C14 | FXC07187- Flag-BamHI-C | gcgaggatccttacttgtcatcgtcatccttgtagtcgcccacagtgatgct |
| Primer-N15 | EcoRV- FXC10490-N | atatgatatcatggggtcgaccgac |
| Primer-C15 | FXC10490- Flag-EcoRI-C | gcgagaattcttacttgtcatcgtcatccttgtagtccacccgctgtatctc |
| Primer-N16 | EcoRV-MGQ-ORK10528 N | atatgatatcatggggcaggcgggctgcaagggg |
| Primer-C16 | ORK10528-Flag-stop-EcoRI | gcgcgaattcttacttacttgtcatcgtcatccttgtagtccgtgctcctgtgggg |
| Primer-N17 | EcoEV-FXC10683-N | atatgatatcatgggctgctgcagc |
| Primer-C17 | FXC10683-Flag-XbaI-C | gcgctctagattacttgtcatcgtcatccttgtagtcagcagaacttgctcc |
| Primer-N18 | EcoRV-FXC11232-N | atgcgatatcatgggagcagtaagc |
| Primer-C18 | FXC11232-Flag-EcoRI-C | gcgcgaattcttacttgtcatcgtcatccttgtagtcacctctctcacccat |
| Primer-N19 | EcoRV- FXC11252-N | gcatgatatcatgggcgggaaccac |
| Primer-C19 | FXC11252- Flag-SacI-C | gcgagagctcttacttgtcatcgtcatccttgtagtcgctgctgcagccacg |
| Primer-C20 | FXC02617- XbaI-C | gcgctctagatattttttcaccact |

**Supplemental Table S1. (4)**

The nucleotide sequences of oligonucleotides used in this study

| Primer | Primer | Sequence |
| --- | --- | --- |
| Primer-N20 | BamHI-FXC2844G2A-N | atatggatccatggccactgtgcacgcccgg |
| Primer-C21 | FXC02940- XbaI-C | gcattctagagatccactgcacaag |
| Primer-N21 | EcoRI-AIF3 N | gcgcgaattcatgggcggctgcttc |
| Primer-C22 | Flag-EcoRV C | gcgcgaattcttacttgtcatcgtc |
| Primer-N22 | BamHI-FXC03534-N | aattggatccatggggagcaaactg |
| Primer-C23 | FXC03969- XbaI-C | gcgctctagacaagttgttattctg |
| Primer-C24 | FXC05945- XbaI-C | gcgctctagaaagcgaagtttcatt |
| Primer-N23 | BamHI- FXC07187-N | gcatggatccatggggaacacattg |
| Primer-C25 | FXC07187- EcoRI-C | gcatgaattcgcccacagtgatgct |
| Primer-C26 | FXC10490- XbaI-C | gatatctagacacccgctgtatctc |
| Primer-N24 | EcoRI-FXC10528-N | atatgaattcatggggcaggcgggc |
| Primer-C27 | FXC10528-XbaI-C | atattctagacgtgctcctgtgggg |
| Primer-N25 | HindIII- FXC10683-N | atgcaagcttatgggctgctgcagc |
| Primer-C28 | FXC10683-XbaI-C | gcgctctagaagcagaacttgctcc |
| Primer-N26 | HindIII- FXC11252-N | gcataagcttatgggcgggaaccac |
| Primer-C29 | FXC11252- EcoRV-C | gatcgatatcgctgctgcagccacg |
