# Supplementary material for: Identification of Human N-Myristoylated Proteins from Human Complementary DNA Resources by Cell-Free and Cellular Metabolic Labeling Analyses
Source: PLoS One. 2015 Aug 26;10(8):e0136360. doi: 10.1371/journal.pone.0136360 (PMC4550359; doi:10.1371/journal.pone.0136360)
Supplement: S2 Table — (DOC) [file pone.0136360.s004.doc]

**Supplemental Table S2.**

| Name of plasmid | Strategy for plasmid construction |
| --- | --- |
| pTD1-Δ10aa-tAct-Flag | Product of a PCR with pDT1-tAct-Flag as a template and primers Primer-N1 and Primer-C1 was EcoRI/XbaI cloned into pTD1. |
| pTD1-FXC01999-FLAG | Product of a PCR with pF1KA0840 as a template and primers Primer-N2 and Primer-C2 was EcoRI/XbaI cloned into pTD1. |
| pTD1-FXC02617-FLAG | Product of a PCR with pF1KB8150 as a template and primers Primer-N3 and Primer-C3 was EcoRV/EcoRI cloned into pTD1. |
| pTD1-FXC02844-FLAG | Product of a PCR with pF1KB8447 as a template and primers Primer-N4 and Primer-C4 was BamHI/XbaI cloned into pTD1. |
| pTD1-FXC02940-FLAG | Product of a PCR with pF1KB8695 as a template and primers Primer-N5 and Primer-C5 was EcoRV/EcoRI cloned into pTD1. |
| pTD1-FXC02961-FLAG | pTD1-FL vector was digested with SgfI and EcoICRI and the KOP cDNA clones digested with SgfI and PmeI were subcloned into the vector. |
| pTD1-FXC03470-FLAG | Product of a PCR with pF1KB6612 as a template and primers Primer-N6 and Primer-C6 was EcoRI/XbaI cloned into pTD1. |
| pTD1-FXC03534-FLAG | Product of a PCR with pF1KE0505 as a template and primers Primer-N7 and Primer-C7 was EcoRV/EcoRI cloned into pTD1. |
| pTD1-FXC03565-FLAG | Product of a PCR with pF1KB5535 as a template and primers Primer-N8 and Primer-C8 was EcoRV/XbaI cloned into pTD1. |
| pTD1-FXC03868-FLAG | Product of a PCR with pF1KB0979 as a template and primers Primer-N9 and Primer-C9 was EcoRI/XbaI cloned into pTD1. |
| pTD1-FXC03969-FLAG | Product of a PCR with pF1KE0678 as a template and primers Primer-N10 and Primer-C10 was EcoRV/EcoRI cloned into pTD1. |
| pTD1-FXC4954-FLAG | Product of a PCR with pF1KB6310 as a template and primers Primer-N11 and Primer-C11 was EcoRI/XbaI cloned into pTD1. |
| pTD1-FXC05856-FLAG | Product of a PCR with pF1KE0523 as a template and primers Primer-N12 and Primer-C12 was EcoRI/XbaI cloned into pTD1. |
| pTD1-FXC05945-FLAG | Product of a PCR with pF1KE0011 as a template and primers Primer-N13 and Primer-C13 was EcoRV/EcoRI cloned into pTD1. |
| pTD1-FXC07187-FLAG | Product of a PCR with pF1KE0451 as a template and primers Primer-N14 and Primer-C14 was EcoRI/BamHI cloned into pTD1. |
| pTD1-FXC10490-FLAG | Product of a PCR with pF1KB9562 as a template and primers Primer-N15 and Primer-C15 was EcoRV/EcoRI cloned into pTD1. |
| pTD1-FXC10528-FLAG | Product of a PCR with pF1KB4832 as a template and primers Primer-N16 and Primer-C16 was EcoRV/EcoRI cloned into pTD1. |
| pTD1-FXC10683-FLAG | Product of a PCR with pF1KB9940 as a template and primers Primer-N17 and Primer-C17 was EcoRV/XbaI cloned into pTD1. |
| pTD1-FXC11232-FLAG | Product of a PCR with pF1KE0683 as a template and primers Primer-N18 and Primer-C18 was EcoRV/EcoRI cloned into pTD1. |
| pTD1-FXC11252-FLAG | Product of a PCR with F1KE0720 as a template and primers Primer-N19 and Primer-C219was EcoRV/SacI cloned into pTD1. |
| pcDNA3-FXC01999-FLAG | Product of a PCR with pF1KA0840 as a template and primers Primer-N2 and Primer-C2 was EcoRI/XbaI cloned into pcDNA3-Flag. |
| pcDNA3-FXC02617-FLAG | Product of a PCR with pF1KB8150 as a template and primers Primer-N3 and Primer-C20 was EcoRV/XbaI cloned into pcDNA3-Flag. |
| pcDNA3-FXC02844-FLAG | Product of a PCR with pF1KB8447 as a template and primers Primer-N4 and Primer-C4 was BamHI/XbaI cloned into pcDNA3-Flag. |
| pcDNA3-FXC02844G2A-FLAG | Product of a PCR with pF1KB8447 as a template and primers Primer-N20 and Primer-C4 was BamHI/XbaI cloned into pcDNA3-Flag. |
| pcDNA3-FXC02940-FLAG | Product of a PCR with pF1KB8695 as a template and primers Primer-N5 and Primer-C21 was EcoRV/XbaI cloned into pcDNA3-Flag. |
| pcDNA3-FXC02961-FLAG | Product of a PCR with pF1KB8718 as a template and primers Primer-N21 and Primer-C22 was EcoRI/EcoRV cloned into pcDNA3-Flag. |
| pcDNA3-FXC03470-FLAG | Product of a PCR with pF1KB6612 as a template and primers Primer-N6 and Primer-C6 was EcoRI/XbaI cloned into pcDNA3-Flag. |
| pcDNA3-FXC03534-FLAG | Product of a PCR with pF1KE0505 as a template and primers Primer-N22 and Primer-C7 was EcoRV/EcoRI cloned into pcDNA3-Flag. |
| pcDNA3-FXC03565-FLAG | Product of a PCR with pF1KB5535 as a template and primers Primer-N8 and Primer-C8 was EcoRV/XbaI cloned into pcDNA3-Flag. |
| pcDNA3-FXC03868-FLAG | Product of a PCR with pF1KB0979 as a template and primers Primer-N9 and Primer-C9 was EcoRI/XbaI cloned into pcDNA3-Flag. |
| pcDNA3-FXC03969-FLAG | Product of a PCR with pF1KE0678 as a template and primers Primer-N10 and Primer-C23 was EcoRV/XbaI cloned into pcDNA3-Flag. |
| pcDNA3-FXC4954-FLAG | Product of a PCR with pF1KB6310 as a template and primers Primer-N11 and Primer-C11 was EcoRI/XbaI cloned into pcDNA3-Flag. |
| pcDNA3-FXC05856-FLAG | Product of a PCR with pF1KE0523 as a template and primers Primer-N12 and Primer-C12 was EcoRI/XbaI cloned into pcDNA3-Flag. |
| pcDNA3-FXC05945-FLAG | Product of a PCR with pF1KE0011 as a template and primers Primer-N13 and Primer-C24 was EcoRV/XbaI cloned into pcDNA3-Flag. |
| pcDNA3-FXC07187-FLAG | Product of a PCR with pF1KE0451 as a template and primers Primer-N23 and Primer-C25 was BamHI/EcoRI cloned into pcDNA3-Flag. |
| pcDNA3-FXC10490-FLAG | Product of a PCR with pF1KB9562 as a template and primers Primer-N15 and Primer-C26 was EcoRV/XbaI cloned into pcDNA3-Flag. |
| pcDNA3-FXC10528-FLAG | Product of a PCR with pF1KB4832 as a template and primers Primer-N24 and Primer-C27 was EcoRI/XbaI cloned into pcDNA3-Flag. |
| pcDNA3-FXC10683-FLAG | Product of a PCR with pF1KB9940 as a template and primers Primer-N25 and Primer-C28 was HindIII/XbaI cloned into pcDNA3-Flag. |
| pcDNA3-FXC11232-FLAG | Product of a PCR with pF1KE0683 as a template and primers Primer-N18 and Primer-C18 was EcoRV/XhoI cloned into pcDNA3-Flag. |
| pcDNA3-FXC11252-FLAG | Product of a PCR with F1KE0720 as a template and primers Primer-N26 and Primer-C29 was HindIII/EcoRV cloned into pcDNA3-Flag. |
