# Supplementary material for: Identification of Human N-Myristoylated Proteins from Human Complementary DNA Resources by Cell-Free and Cellular Metabolic Labeling Analyses
Source: PLoS One. 2015 Aug 26;10(8):e0136360. doi: 10.1371/journal.pone.0136360 (PMC4550359; doi:10.1371/journal.pone.0136360)
Supplement: S3 Table — (DOCX) [file pone.0136360.s005.docx]

| No. | FXC No. | Gene name | MYR Predictor | Myristoylator |
| --- | --- | --- | --- | --- |
| 46 | FXC04325 | NPAP1 | Reliable | High Confidence |
| 47 | FXC04954 | FAM131C | Reliable | High Confidence |
| 48 | FXC05230 | NCS1 | Reliable | High Confidence |
| 49 | FXC05310 | GNAI2 | Reliable | High Confidence |
| 50 | FXC05311 | GNAI3 | Reliable | High Confidence |
| 51 | FXC05451 | HPCAL4 | Reliable | High Confidence |
| 52 | FXC05791 | KRTAP5-2 | Twilight | No |
| 53 | FXC05792 | KRTAP5-3 | Twilight | No |
| 54 | FXC05793 | KRTAP5-4 | Twilight | No |
| 55 | FXC05856 | DRICH1 | Twilight | High Confidence |
| 56 | FXC05932 | MARCKSL1 | Reliable | High Confidence |
| 57 | FXC05945 | MCC1 | No | High Confidence |
| 58 | FXC06109 | NCALD | Reliable | High Confidence |
| 59 | FXC06445 | PPEF2 | Reliable | High Confidence |
| 60 | FXC06481 | PRKACG | Reliable | High Confidence |
| 61 | FXC06491 | PRKG2 | Reliable | Medium Confidence |
| 62 | FXC07187 | TOMM40L | Twilight | Medium Confidence |
| 63 | FXC07438 | CBX1 | No | High Confidence |
| 64 | FXC07768 | CHCHD3 | Reliable | High Confidence |
| 65 | FXC07771 | ZNF292 | No | High Confidence |
| 66 | FXC07968 | ARL5A | No | High Confidence |
| 67 | FXC08024 | RCVRN | Reliable | High Confidence |
| 68 | FXC08348 | GORASP2 | Reliable | High Confidence |
| 69 | FXC10143 | VSNL1 | Reliable | High Confidence |
| 70 | FXC10490 | HID1 | No | High Confidence |
| 71 | FXC10528 | P2RX5 | Reliable | High Confidence |
| 72 | FXC10564 | PRKACA | Reliable | High Confidence |
| 73 | FXC10600 | DUSP22 | No | High Confidence |
| 74 | FXC10609 | ARF3 | Twilight | High Confidence |
| 75 | FXC10612 | CIB2 | Reliable | High Confidence |
| 76 | FXC10616 | ARF5 | Reliable | High Confidence |
| 77 | FXC10683 | SLC44A1 | Reliable | High Confidence |
| 78 | FXC10684 | FYN | Reliable | High Confidence |
| 79 | FXC10889 | KCNJ2 | Twilight | No |
| 80 | FXC10897 | CHP2 | Twilight | High Confidence |
| 81 | FXC10914 | CABP2 | No | Medium Confidence |
| 82 | FXC11186 | KRTAP5-7 | Twilight | No |
| 83 | FXC11187 | KRTAP5-1 | Twilight | No |
| 84 | FXC11232 | SGK494 | Reliable | No |
| 85 | FXC11252 | STK32B | Twilight | High Confidence |
| 86 | FXC11260 | PSKH1 | Reliable | High Confidence |
| 87 | FXC11288 | OR9K1P | No | Medium Confidence |
| 88 | FXC11366 | SELL | Twilight | No |
| 89 | FXC11854 | SRC | Reliable | High Confidence |
| 90 | FXC11932 | KIAA1586 | No | High |

**Supplemental Table S3.**

| No. | FXC No. | Gene name | MYR Predictor | Myristoylator |
| --- | --- | --- | --- | --- |
| 1 | FXC01999 | FBXL7 | Twilight | No |
| 2 | FXC02047 | CCSER1 | No | Low Confidence |
| 3 | FXC02078 | RPL18 | No | Low Confidence |
| 4 | FXC02087 | PSMC1 | Reliable | High Confidence |
| 5 | FXC02096 | MBP | Twilight | High Confidence |
| 6 | FXC02112 | GNAO1 | Reliable | High Confidence |
| 7 | FXC02124 | ARF6 | Reliable | High Confidence |
| 8 | FXC02148 | GNAZ | Reliable | High Confidence |
| 9 | FXC02199 | HPCAL1 | Reliable | High Confidence |
| 10 | FXC02452 | MLNR | No | Medium Confidence |
| 11 | FXC02617 | PPM1B | No | Medium Confidence |
| 12 | FXC02691 | ARF4 | Reliable | High Confidence |
| 13 | FXC02705 | CIB1 | Reliable | High Confidence |
| 14 | FXC02814 | ZNRF1 | Reliable | No |
| 15 | FXC02819 | SOX6 | No | High Confidence |
| 16 | FXC02830 | SCYL3 | Reliable | High Confidence |
| 17 | FXC02835 | PRKACB | Reliable | High Confidence |
| 18 | FXC02843 | CYB5R3 | Reliable | High Confidence |
| 19 | FXC02844 | SAMM50 | No | Medium Confidence |
| 20 | FXC02903 | CCNY | Reliable | High Confidence |
| 21 | FXC02940 | PLEKHN isoform2 | Reliable | Low Confidence |
| 22 | FXC02961 | AIFM3 | Reliable | High Confidence |
| 23 | FXC02992 | ARL11 | Reliable | Medium Confidence |
| 24 | FXC02995 | ARL4A | No | High Confidence |
| 25 | FXC02996 | ARL4D | No | Low Confidence |
| 26 | FXC03133 | ARL4C | Reliable | Low Confidence |
| 27 | FXC03149 | MARCKS | Reliable | High Confidence |
| 28 | FXC03406 | KCNIP1 | Reliable | No |
| 29 | FXC03442 | FRS2 | Reliable | High Confidence |
| 30 | FXC03444 | LYN | Reliable | High Confidence |
| 31 | FXC03470 | PAG1 | No | High Confidence |
| 32 | FXC03513 | GORASP1 | Reliable | High Confidence |
| 33 | FXC03534 | C22orf42 | Twilight | High Confidence |
| 34 | FXC03565 | CTSC | No | Low Confidence |
| 35 | FXC03640 | MMP15 | No | High Confidence |
| 36 | FXC03679 | SLA2 | Reliable | High Confidence |
| 37 | FXC03685 | PDE9A | Twilight | High Confidence |
| 38 | FXC03736 | LCK | Reliable | High Confidence |
| 39 | FXC03765 | YAF2 | No | High Confidence |
| 40 | FXC03808 | BLK | Reliable | High Confidence |
| 41 | FXC03815 | RAPSN | Reliable | High Confidence |
| 42 | FXC03868 | RNF141 | No | High Confidence |
| 43 | FXC03887 | PPP3R1 | Reliable | High Confidence |
| 44 | FXC03909 | GMCL1 | Twilight | High Confidence |
| 45 | FXC03969 | STK32A | Twilight | High |
