# Supplementary material for: Identification of Human N-Myristoylated Proteins from Human Complementary DNA Resources by Cell-Free and Cellular Metabolic Labeling Analyses
Source: PLoS One. 2015 Aug 26;10(8):e0136360. doi: 10.1371/journal.pone.0136360 (PMC4550359; doi:10.1371/journal.pone.0136360)
Supplement: S5 Table — (DOCX) [file pone.0136360.s007.docx]

**Supplemental Table S5.**

| Lane | FXC No. | Gene name |
| --- | --- | --- |
| 1 | FXC01999 | FBXL7 |
| 2 | FXC02617 | PPM1B |
| 3 | FXC02844 | SAMM50 |
| 4 | FXC02940 | PLEKHN |
| 5 | FXC02961 | AIFM3 |
| 6 | FXC03470 | PAG1 |
| 7 | FXC03534 | C22orf42 |
| 8 | FXC03565 | CTSC |
| 9 | FXC03868 | RNF141 |
| 10 | FXC03969 | STK32A |
| 11 | FXC04954 | FAM131C |
| 12 | FXC05856 | DRICH1 |
| 13 | FXC05945 | MCC1 |
| 14 | FXC07187 | TOMM40L |
| 15 | FXC10490 | HID1 |
| 16 | FXC10528 | P2RX5 |
| 17 | FXC10683 | SLC44A1 |
| 18 | FXC11232 | SGK494 |
| 19 | FXC11252 | STK32B |
